# Theta Burst Stimulation Protocols for Schizophrenia: A Systematic Review and Network Meta-Analysis

**DOI:** 10.1001/jamanetworkopen.2024.41159

**Published:** 2024-10-24

**Authors:** Taro Kishi, Toshikazu Ikuta, Kenji Sakuma, Shun Hamanaka, Yasufumi Nishii, Masakazu Hatano, Shinsuke Kito, Nakao Iwata

**Affiliations:** 1 Department of Psychiatry, Fujita Health University School of Medicine, Toyoake, Aichi, Japan; 2 Department of Communication Sciences and Disorders, School of Applied Sciences, University of Mississippi, Oxford; 3 Department of Pharmacotherapeutics and Informatics, Fujita Health University School of Medicine, Toyoake, Aichi, Japan; 4 Department of Psychiatry, Jikei University School of Medicine, Minato-ku, Tokyo, Japan

## Abstract

**Importance:**

To date, several theta burst stimulation (TBS) protocols, such as intermittent TBS (iTBS), have been proposed; however, previous systematic reviews have revealed inconsistent efficacy findings in individual TBS studies for schizophrenia.

**Objective:**

To examine which TBS protocols are associated with the most favorable and acceptable outcomes in adults with schizophrenia.

**Data Sources:**

The Cochrane Library, PubMed, and Embase databases were searched for studies published before May 22, 2024.

**Study Selection:**

The inclusion criteria were as follows: (1) published and unpublished randomized clinical trials (RCTs) of any TBS treatment and (2) RCTs including individuals with schizophrenia spectrum disorders, other psychotic disorders, or both.

**Data Extraction and Synthesis:**

This study followed the Cochrane standards for data extraction and data quality assessment and used the Preferred Reporting Items for Systematic Reviews and Meta-Analyses guideline for reporting. The risk of bias of individual studies was assessed using the second version of the Cochrane risk of bias tool, and the Confidence in Network Meta-Analysis application was used to rate the certainty of evidence for meta-analysis results. At least 2 authors double-checked the literature search, data transfer accuracy, and calculations.

**Main Outcomes and Measures:**

The primary outcome of this study was improvement in scores related to negative symptoms. Our frequentist network meta-analysis used a random-effects model. The standardized mean difference (SMD) or odds ratio for continuous or dichotomous variables, respectively, was calculated with 95% CIs.

**Results:**

A total of 30 RCTs of 9 TBS protocols, with 1424 participants, were included. Only iTBS over the left dorsolateral prefrontal cortex (L-DLPFC) was associated with reduced negative symptom scores (SMD, −0.89; 95% CI, −1.24 to −0.55), overall symptom scores (SMD, −0.81; 95% CI, −1.15 to −0.48), Positive and Negative Syndrome Scale general subscale scores (SMD, −0.57; 95% CI, −0.89 to −0.25), depressive symptom scores (SMD, −0.70; 95% CI, −1.04 to −0.37), and anxiety symptom scores (SMD, −0.58; 95% CI, −0.92 to −0.24) and improved overall cognitive impairment scores (SMD, −0.52; 95% CI, −0.89 to −0.15) compared with a sham. However, positive symptom score changes, all-cause discontinuation rate, discontinuation rate due to adverse events, headache incidence, and dizziness incidence did not significantly differ between any TBS protocols and sham.

**Conclusions and Relevance:**

In this network meta-analysis, iTBS over the L-DLPFC was associated with improved scores for negative, depressive, anxiety, and cognitive symptoms in individuals with schizophrenia and was well tolerated by the participants. Other forms of TBS were not associated with benefit. Further research is needed to assess the potential role of TBS in the treatment of schizophrenia.

## Key Points

**Question:**

Which theta burst stimulation (TBS) protocols are associated with the most effective and acceptable outcomes in adults with schizophrenia?

**Findings:**

This systematic review and network meta-analysis including 30 randomized sham-controlled trials with 1424 participants found that intermittent TBS (iTBS) over the left-dorsolateral prefrontal cortex (L-DLPFC) was associated with reduced negative symptom scores, overall symptom scores, Positive and Negative Syndrome Scale general subscale scores, depressive symptom scores, and anxiety symptom scores and improved overall cognitive impairment scores compared with a sham. Rates of treatment discontinuation did not differ among treatment protocols or sham.

**Meaning:**

The findings of this study suggest that iTBS over the L-DLPFC may be a useful treatment for symptoms associated with schizophrenia.

## Teaser

This systematic review and meta-analysis examines which theta burst stimulation protocols are associated with symptom improvements and tolerability among adults with schizophrenia.

## Introduction

Schizophrenia, a severe mental illness affecting approximately 1% of the population, is characterized by positive, negative, cognitive, and affective symptoms.^1^ Antipsychotics exhibited beneficial effects on positive symptoms in individuals with typical schizophrenia; however, they are less effective against negative symptoms or cognitive dysfunction.^2^ Thus, research is warranted to investigate the existence of therapeutic targets other than dopamine receptors for treating negative and depressive symptoms and cognitive decline.

Recently, repetitive transcranial magnetic stimulation (rTMS) has attracted global attention as a therapeutic tool for various neurological and psychiatric conditions. The US Food and Drug Administration approved rTMS, which is a noninvasive therapeutic brain stimulation technique for modulating the regional excitability of the human brain for major depressive disorder (MDD), obsessive-compulsive disorder, and smoking cessation.^3^ rTMS targeted at the left dorsomedial prefrontal cortex (L-DLPFC) increases activity in the L-DLPFC, which is underactive in individuals with MDD, and promotes therapeutic connections with the anterior cingulate and amygdala, which play a crucial role in coordinating stress responses.^4,5,6^ The DLPFC is closely connected to the orbitofrontal cortex, thalamus, parts of the basal ganglia, hippocampus, and primary and secondary association areas of the neocortex, which are related to the pathophysiology of schizophrenia.^4,7^ Moreover, the DLPFC is involved in reward processing and cognition, including executive function, working memory, and spatial attention.^7^ Therefore, this region is an attractive target for treating not only negative and depressive symptoms but also cognitive decline in individuals with schizophrenia. Recent systematic review articles have revealed that numerous rTMS trials for schizophrenia have been conducted.^8,9,10,11,12,13,14,15,16,17,18^

Theta burst stimulation (TBS), a new noninvasive therapeutic brain stimulation technique, has been developed.^3^ In general, TBS is delivered over a much shorter time than conventional rTMS.^19,20^ A recent randomized clinical trial (RCT) found that intermittent TBS (iTBS) over the L-DLPFC exerted similar effects on MDD as conventional high-frequency rTMS over the L-DLPFC.^21^ Thus, iTBS over the L-DLPFC could be a more practical and potentially more efficient therapeutic modality. To date, several other TBS protocols, such as continuous TBS (cTBS), have been proposed, along with iTBS over the L-DLPFC (eTable 1 in Supplement 1). Previous systematic reviews^8,9,10,11,12,13,14,15,16,17,18^ have revealed inconsistent results regarding the efficacy of individual TBS for schizophrenia. Therefore, we conducted a systematic review and random-effects model network meta-analysis on 11 outcomes related to the efficacy, acceptability, tolerability, and safety of 9 TBS protocols for treating adults with schizophrenia. Because potential modifiers associated with TBS efficacy in patients with schizophrenia remain unknown, we attempted to determine variables in the participants, treatment, and/or study design that could affect the effect size for the primary outcome in the pairwise meta-regression analyses.

## Methods

This study was conducted under the Preferred Reporting Items for Systematic Reviews and Meta-Analyses (PRISMA) reporting guideline^22,23^ and was registered in the Open Science Framework.^24^ At least 2 authors double-checked the literature search, data transfer accuracy, and calculations.

### Inclusion Criteria, Exclusion Criteria, and Search Strategy

The inclusion criteria were as follows: (1) published and unpublished RCTs of any TBS treatment and (2) RCTs including individuals with schizophrenia spectrum disorders, other psychotic disorders, or both. The exclusion criteria were as follows: (1) RCTs including individuals comorbid with substance use disorders and (2) RCTs involving children or adolescents with the aforementioned disorders. We searched the Cochrane Library, PubMed, and Embase databases for studies published before May 22, 2024. The eFigure in Supplement 1 shows detailed information regarding the search strategy.

### Outcome Measures, Data Synthesis, and Data Extraction

The primary outcome of this study was improvement in scores related to negative symptoms (Scale for the Assessment of Negative Symptoms [SANS] and Positive and Negative Syndrome Scale [PANSS] negative subscale scores), overall symptoms (PANSS total scores), positive symptoms (Scale for the Assessment of Positive Symptoms and PANSS positive subscale scores), PANSS general subscale scores, depressive symptoms (Calgary Depression Scale for Schizophrenia, Hamilton Depression Rating Scale, and Apathy Evaluation Scale scores), anxiety symptoms (Hamilton Anxiety Rating Scale scores), and overall cognitive function (Schizophrenia Cognition Rating Scale, Montreal Cognitive Assessment Test, and Measurement and Treatment Research to Improve Cognition in Schizophrenia [MATRICS] Consensus Cognitive Battery scores), all-cause discontinuation rate, discontinuation rate due to adverse events, headache incidence, and dizziness occurrence. eTable 2 in Supplement 1 presents the data synthesis for efficacy outcomes. We conducted a meta-analysis of the outcomes, which included at least 4 RCTs. The extracted data were analyzed according to the intention-to-treat or modified intention-to-treat principles. However, completer analysis data were not excluded to obtain as much information as possible. We searched for data in published systematic review articles if the required data were missing. Furthermore, we attempted to contact the original investigators to obtain unpublished data.

### Statistical Analysis

This frequentist network meta-analysis used a random-effects model.^25,26^ The standardized mean difference (SMD) or odds ratio for continuous or dichotomous variables, respectively, was calculated with 95% CIs. Heterogeneity was assessed using τ^2^ statistics and *I*^2^ statistics.^27^ A statistical evaluation of incoherence was impossible because no head-to-head studies have compared different TBS protocols in the trials included in our meta-analysis. The surface under the curve cumulative ranking probabilities were used to rank the treatments for each outcome. We identified the sufficiency of the distribution differences to validate the analysis by comparing the distribution of possible effect modifiers across included treatments in the network meta-analysis using the Kruskal-Wallis test (continuous variables) and the Pearson χ^2^ test or Fisher exact test (categorical variables) and by assessing their actual influence on the treatment effect through network meta-regression analyses (eTable 3 in Supplement 1). Potential confounding factors included individuals with predominantly negative symptoms (studies including individuals with predominantly negative symptoms vs other studies), female proportion, mean age, total number of participants, antipsychotic dose,^28^ coil localization and targeting method (studies using magnetic resonance imaging [MRI] vs studies not using MRI), TBS coil (studies using figure-8 coils vs studies using circulator coils), use of sham coils (studies using sham coils vs studies not using sham coils), percentage motor threshold, number of sessions during a day, number of sessions during a trial, number of pulses during a session, number of pulses during a trial, negative symptoms scales (studies using SANS vs studies using PANSS), publication year, country where the trial was conducted (studies conducted in China vs studies conducted in other countries), and overall risk of bias (low risk or some concerns studies vs high-risk studies). Version 2 of the Cochrane risk of bias tool for RCTs^29^ was used to evaluate the overall risk of bias for every RCT. Furthermore, we performed a sensitivity analysis for the primary outcome, excluding studies whose overall risk of bias was high. Moreover, we performed a subgroup analysis involving only trials that included individuals with predominantly negative symptoms as the primary outcome. Finally, the results were incorporated into the Confidence in Network Meta-Analysis application, which is an adaptation of the Grading of Recommendations Assessment, Development, and Evaluation approach, to evaluate the credibility of the results of each network meta-analysis.^30^

We conducted pairwise meta-regression analyses to investigate the association of the differences in the characteristics of the participants, treatment, and/or study design with the effect size for the primary outcome in iTBS over the L-DLPFC, which was the only treatment superior to sham in our network meta-analysis. To perform this meta-regression analysis, a random-effects model pairwise meta-analysis was performed.^25^ This pairwise meta-regression considered the same factors involved in the network meta-regression. Moreover, we added an intersession interval between the TBS treatments as a factor in this pairwise meta-regression. Comprehensive Meta-Analysis Software version 3 (Biostat Inc) was used for pairwise meta-analysis and pairwise meta-regression. A 2-sided *P* < .05 denotes statistical significance.

## Results

### Study Characteristics

The eFigure in Supplement 1 presents the literature search and a detailed explanation of the process. Initially, 198 articles were identified, of which 65 were duplicates, 112 articles were excluded after title and abstract screening, and 3 were excluded after full-text review. An additional 12 studies were found from previous review articles.^8,9,10,11,12,13,14,15,16,17,18^ Finally, this systematic review included 30 RCTs with 1424 participants (mean [SD] age, 40.5years; 44.3% male).^31,32,33,34,35,36,37,38,39,40,41,42,43,44,45,46,47,48,49,50,51,52,53,54,55,56,57,58,59,60^ Twenty-six studies (86.7%) included only individuals with schizophrenia,^32,33,34,35,36,37,38,39,40,41,42,43,44,45,46,47,48,51,52,54,55,56,57,58,59,60^ 10 studies (33.3%) included individuals with predominantly negative symptoms,^43,44,32,34,35,50,36,37,54,58^ and 19 studies (63.3%) used a sham coil as a control.^43,44,31,45,32,33,46,35,49,50,51,52,53,55,54,56,57,38,39^ The following 9 TBS protocols and target regions were assessed: cTBS over the left primary motor cortex, cTBS over the left temporoparietal cortex, cTBS over the left and right temporoparietal cortex, cTBS over the right inferior parietal lobule, iTBS over the cerebellar vermis (CV), iTBS over the L-DLPFC, iTBS over the left inferior frontal gyrus, iTBS over the left supplementary motor area, and iTBS over the right DLPFC. However, our meta-analysis excluded iTBS over the left inferior frontal gyrus because data on this TBS treatment were unavailable. Eighteen studies (60.0%) used iTBS over the L-DLPFC.^32,33,34,35,36,37,38,39,40,41,42,44,46,53,56,57,58,59^ eTable 1 in Supplement 1 presents the study characteristics. For the overall risk of bias, 17 studies (56.7%) were evaluated as having some concerns^32,33,35,36,37,40,43,44,46,47,48,49,50,53,57,58,59^ (eTable 4 in Supplement 1). Significant differences in the number of sessions during a trial, number of pulses during a trial, and country where the trial was conducted were observed among the TBS protocols (eTable 3 in Supplement 1).

### Network Meta-Analysis Results

**Figure 1.**
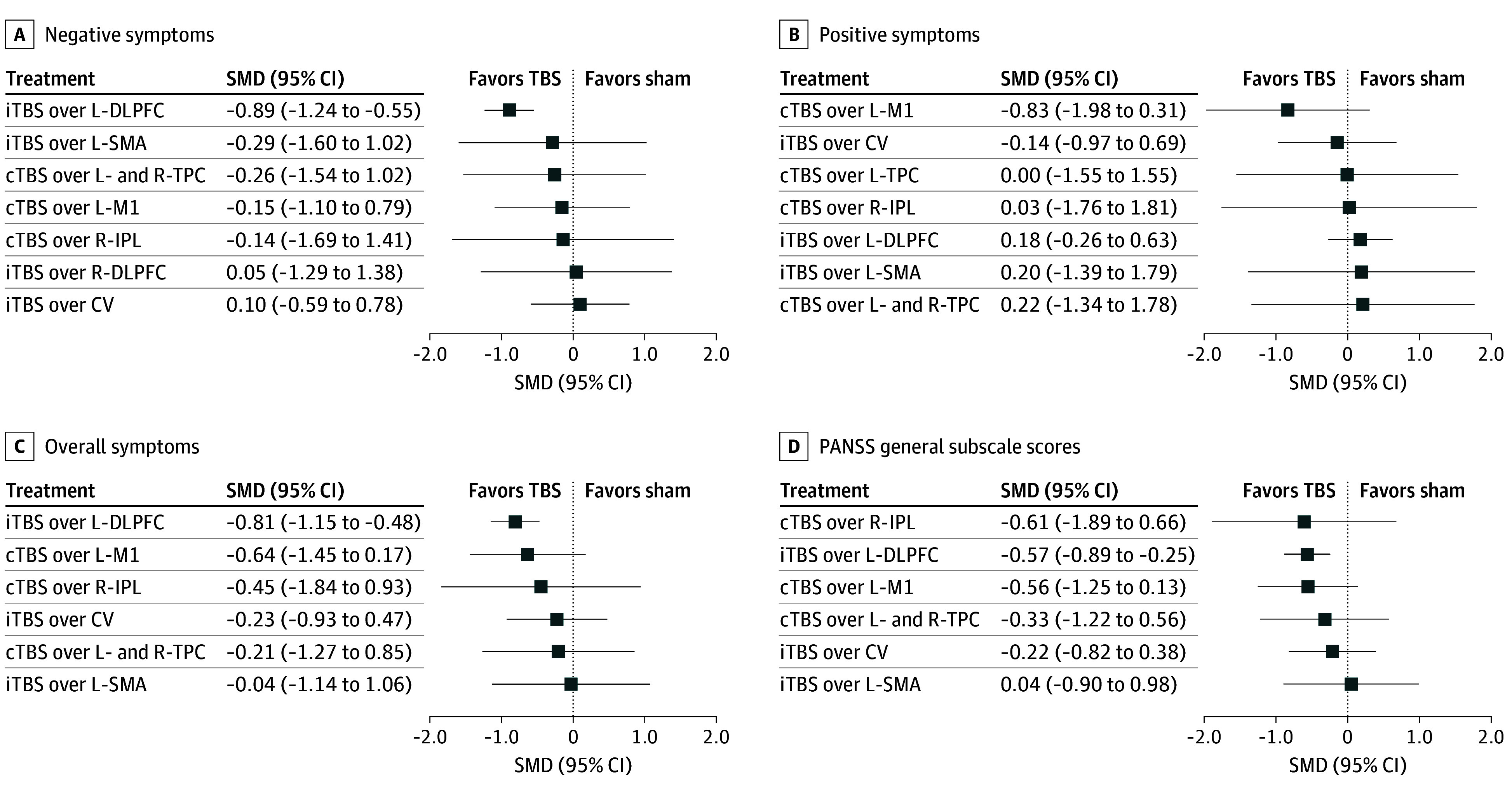
Forest Plots for Negative, Positive, and Overall Symptoms and Positive and Negative Syndrome Scale (PANSS) General Subscale Active treatments were compared with sham treatments. cTBS indicates continuous theta burst stimulation; CV, cerebellar vermis; iTBS, intermittent theta burst stimulation; L-DLPFC, left dorsolateral prefrontal cortex; L-M1, left primary motor cortex; L-SMA, left supplementary motor area, L-TPC, left temporoparietal cortex; R-DLPFC, right dorsolateral prefrontal cortex; R-IPL, right inferior parietal lobule; R-TPC, right tempoparietal cortex; and SMD, standardized mean difference.

iTBS over the L-DLPFC exhibited a significantly greater reduction in negative symptom scores than a sham (SMD, −0.89; 95% CI, −1.24 to −0.55) (Figure 1; eAppendix 1 in Supplement 1). Furthermore, iTBS over the L-DLPFC was superior to iTBS over the CV (SMD, −0.99; 95% CI, −1.76 to −0.22) (eAppendix 1 in Supplement 1).

Global heterogeneity was assessed as high. At least 10 studies included no comparisons other than iTBS over the L-DLPFC; however, the funnel plots of the primary outcome revealed symmetry (eAppendix 1 in Supplement 1). The network meta-regression analyses revealed no potential confounding factors associated with the effect size of the primary outcome (eAppendix 1 in Supplement 1). Compared with a sham, the effect size for each TBS protocol on the primary analysis was similar to that of not only the sensitivity analysis but also the subgroup analysis (eAppendix 1 in Supplement 1). However, the primary analysis resulted in a smaller effect size for iTBS over the L-DLPFC than the sensitivity analysis (SMD, −1.03; 95% CI, −1.45 to −0.62) and the subgroup analysis (SMD, −1.27; 95% CI, −2.14 to −0.40). The estimated between-study variance for these analyses was similar.

**Figure 2.**
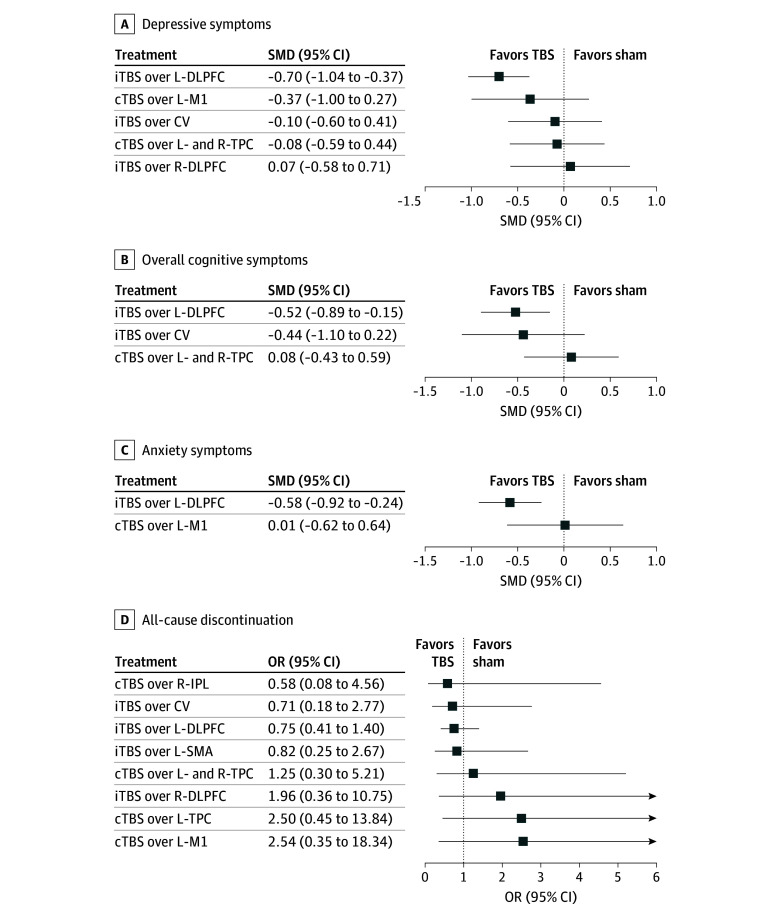
Forest Plots for Depressive, Overall Cognitive, and Anxiety Symptoms and All-Cause Discontinuation B, The algebraic sign of the numerical scores for overall cognitive symptoms was reversed because lower scores indicate a higher impairment. Active treatments were compared with the sham. cTBS indicates continuous theta burst stimulation; CV, cerebellar vermis; iTBS, intermittent theta burst stimulation; L-DLPFC, left dorsolateral prefrontal cortex; L-M1, left primary motor cortex; L-SMA, left supplementary motor area; L-TPC, left temporoparietal cortex; OR, odds ratio; R-DLPFC, right dorsolateral prefrontal cortex; R-IPL, right inferior parietal lobule; R-TPC, right tempoparietal cortex; and SMD, standardized mean difference.

iTBS over the L-DLPFC was associated with reduced overall symptom scores (SMD, −0.81; 95% CI, −1.15 to −0.48) (Figure 1; eAppendix 2 in Supplement 1), PANSS general subscale scores (SMD, −0.57; 95% CI, −0.89 to −0.25) (Figure 1; eAppendix 4 in Supplement 1), depressive symptom scores (SMD, −0.70; 95% CI, −1.04 to −0.37) (Figure 2; eAppendix 5 in Supplement 1), and anxiety symptom scores (SMD,−0.58; 95% CI, −0.92 to −0.24) (Figure 2; eAppendix 6 in Supplement 1) and improved overall cognitive function scores (SMD, −0.52; 95% CI, −0.89 to −0.15) (Figure 2; eAppendix 7 in Supplement 1) compared with a sham. However, no significant differences were observed in changes in the positive symptom score (Figure 1; eAppendix 3 in Supplement 1), all-cause discontinuation rate (Figure 2; eAppendix 8 in Supplement 1), discontinuation rate due to adverse events (eAppendix 9 in Supplement 1), headache incidence (eAppendix 10 in Supplement 1), and dizziness occurrence (eAppendix 11 in Supplement 1) between any of the TBS protocols and a sham.

Global heterogeneity for the improvement in overall symptom scores, positive symptom scores, and PANSS general subscale scores was assessed as moderate to high or high. The within-study bias of most comparisons was assessed as some concerns or high risk. Moreover, funnel plots with fewer than 10 studies were not meaningful^27^; thus, all comparisons vs a sham for publication bias were assessed as some concerns. Additionally, the comparison was downgraded one level if only indirect evidence was available. Consequently, confidence in the evidence was generally assessed as low or very low.

### Pairwise Meta-Analysis Results

Compared with a sham, iTBS over the L-DLPFC was associated with a significant improvement in negative symptom scores (SMD, −0.94; 95% CI, −1.33 to −0.54; *I*^2^ = 82.7%) (eAppendix 1 in Supplement 1). The pairwise meta-regression analysis revealed that studies that included individuals who received a higher antipsychotic dose were associated with a larger effect size for the improvement in negative symptom scores than studies that included individuals who received a lower antipsychotic dose (eAppendix 1 in Supplement 1). Studies with more pulses during a trial were associated with a larger effect size for the primary outcome than studies with fewer pulses during a trial (eAppendix 1 in Supplement 1). Studies that used the SANS had a larger effect size for the primary outcome than studies that used the PANSS (eAppendix 1 in Supplement 1).

## Discussion

To our knowledge, this is the first systematic review and network meta-analysis to compare the efficacy, acceptability, tolerability, and safety of various TBS treatment protocols in individuals with schizophrenia. Our results revealed that iTBS over the L-DLPFC was associated with a benefit for treating schizophrenia, particularly for negative, depressive, and anxiety symptoms and cognitive impairment. Moreover, iTBS over the L-DLPFC had good acceptability, tolerability, and safety profiles in individuals with schizophrenia. However, clinicians must monitor patients, although rTMS rarely induces seizures.^61^ Meanwhile, other TBS protocols exhibited no association with schizophrenia symptom improvements. However, because of the small number of participants and studies on TBS protocols other than iTBS over the L-DLPFC in our meta-analysis, larger studies are warranted to generate robust evidence.

In individuals with schizophrenia, iTBS over the L-DLPFC appears to improve negative, depressive, and anxiety symptoms and cognitive impairment. Negative symptoms are primary or secondary to depression or overlap with depressive symptoms. Anhedonia and psychomotor retardation in schizophrenia are considered depressive and negative symptoms.^62^ Moreover, negative symptoms are associated with neurocognitive symptoms.^63^ Previous meta-analyses have revealed that iTBS over the L-DLPFC was associated with improvements in depressive symptoms in individuals with mood disorders.^10,19^ These results suggest that iTBS over the L-DLPFC is not specifically effective against negative and depressive symptoms of schizophrenia but is effective against these symptoms experienced by individuals with various psychiatric disorders across diseases. Therefore, the therapeutic effects of iTBS over the L-DLPFC may be specific to symptoms but not diagnostic categories.^64^ Hypofrontality has been indicated for the pathophysiology of schizophrenia and mood disorders.^64^ Several studies have reported that glutamate signaling imbalance in the prefrontal cortex may account for these symptoms in individuals with schizophrenia and mood disorders.^65^ A recent meta-analysis of schizophrenia revealed dysfunctional regulation (ie, increased variability) of glutamatergic metabolite concentrations, particularly in the DLPFC.^66^ Furthermore, rTMS noninvasively modulated cortical excitability directly in targeted cortical areas and their associated networks. By reducing abnormal neurotransmission in the brain neural network mediated by the L-DLPFC, iTBS over the L-DLPFC may improve these symptoms.

Our pairwise meta-regression analysis for iTBS over the L-DLPFC found that studies that included individuals who received higher antipsychotic doses had a larger effect size for negative symptom score improvement than studies that included individuals who received lower antipsychotic doses. Most studies included in our systematic review did not report the severity of extrapyramidal symptoms; however, patients who received high antipsychotic doses may have experienced extrapyramidal symptoms. Negative symptoms in schizophrenia can be categorized as primary or secondary.^67^ Primary negative symptoms are intrinsic to schizophrenia, whereas secondary negative symptoms are related to other factors, such as medication adverse effects (ie, extrapyramidal symptoms).^67^ Recently, several studies have revealed that rTMS over the L-DLPFC may be effective for depression, anxiety, and motor symptoms in patients with Parkinson disease.^68,69^ Therefore, rTMS may improve secondary negative symptoms in individuals with schizophrenia caused by reduced extrapyramidal symptoms. Further studies investigating antipsychotic-related extrapyramidal symptom improvement with iTBS over the L-DLPFC are warranted.

One of our hypotheses was an association between the number of pulses administered and greater antipsychotic effect. Our pairwise meta-regression analysis for iTBS over the L-DLPFC found that studies with more pulses during a trial had larger effect sizes for the primary outcome than those with fewer pulses during a trial, which supports our hypothesis. Recently, a new iTBS over the L-DLPFC treatment protocol for MDD, named Stanford neuromodulation therapy (SNT), was developed.^70^ The SNT protocol comprises 10 iTBS over the L-DLPFC sessions, with a total of 18 000 pulses daily, for 5 consecutive days.^70^ An RCT of SNT revealed that iTBS over the L-DLPFC outperformed sham in improving depressive symptoms with a large effect size (Cohen *d* >0.8).^70^ The development of such an accelerated iTBS over the L-DLPFC protocol for schizophrenia is required.^71^

This pairwise meta-regression analysis for iTBS over the L-DLPFC found that studies that used the SANS had a larger effect size for the primary outcome than those that used the PANSS. A high correlation was observed between SANS and PANSS negative symptom ratings.^72^ Among the trials included in our meta-analysis, only 6 used the SANS. Therefore, we could not discuss this association in depth.

Recent meta-analyses have revealed that electroconvulsive therapy (ECT), another neuromodulation therapy, improved positive symptoms but not negative symptoms in patients with treatment-resistant schizophrenia who received nonclozapine antipsychotic medications^73^ and those with clozapine-resistant schizophrenia.^74^ A recent MRI study found that ECT may reduce positive psychotic symptoms in patients with schizophrenia by preferentially targeting limbic brain areas, such as the parahippocampal gyrus/hippocampus.^75^ These results indicate that TMS, which stimulates deeper brain areas, including the limbic system, might improve positive symptoms in individuals with schizophrenia. A novel treatment that exhibited a benefit for positive and negative symptoms may be developed by elucidating the differences in the therapeutic mechanisms between ECT and iTBS over the L-DLPFC.

### Limitations

Our study has several limitations that must be considered. First, our meta-analysis included a small number of participants and studies. Second, the study participants included in the meta-analysis received various antipsychotics and other psychotropic drugs. In particular, benzodiazepine may inhibit rTMS response, whereas psychostimulant use may increase rTMS response.^76,77^ Third, efficacy data from the day closest to TMS treatment completion were used. The effect size may be greater over a longer period if the antipsychotic effect in the TBS treatment group persists and the antipsychotic effect in the sham group is attenuated. Consequently, larger-scale, long-term studies on TBS protocols are required to evaluate the longevity of their effects (eg, through continuation studies). Fourth, our study could not evaluate the characteristics of patients (eg, treatment-resistant schizophrenia) who would benefit from iTBS over the L-DLPFC because most RCTs included in our systematic review involved patients with various characteristics. Furthermore, our study did not consider several factors for informed choices in daily clinical practice, such as pharmacotherapy integration, other nonpharmacological interventions, and cost-effectiveness analysis.

## Conclusions

In this systematic review and network meta-analysis, iTBS over the L-DLPFC was associated with improved scores for negative, depressive, anxiety, and cognitive symptoms in individuals with schizophrenia. Additionally, it was well tolerated by participants. These findings suggest that iTBS over the L-DLPFC has the potential to become a novel treatment for individuals with schizophrenia. A large-scale randomized clinical trial is needed to confirm this conclusion.

## Notes

Supplement 1. **eFigure.** Study Flow Diagram
eReferences.
**eTable 1.** Characteristics of the Randomized Clinical Trials in Our Systematic Review **eTable 2.** Data Synthesis **eTable 3.** Transitivity Assessment **eTable 4.** Risk of Bias Summary **eAppendix 1.** Negative Symptoms **eAppendix 2.** Overall Symptoms **eAppendix 3.** Positive Symptoms **eAppendix 4.** PANSS General Subscale Score **eAppendix 5.** Depressive Symptoms **eAppendix 6.** Anxiety Symptoms **eAppendix 7.** Overall Cognitive Impairment **eAppendix 8.** All-Cause Discontinuation **eAppendix 9.** Discontinuation Due to Adverse Events **eAppendix 10.** Headache **eAppendix 11.** Dizziness

Supplement 2.
Data Sharing Statement
