# Supplementary material for: Theta Burst Stimulation Protocols for Schizophrenia: A Systematic Review and Network Meta-Analysis
Source: JAMA Netw Open. 2024 Oct 24;7(10):e2441159. doi: 10.1001/jamanetworkopen.2024.41159 (PMC11581676; doi:10.1001/jamanetworkopen.2024.41159)
Supplement: Supplement 1. — eFigure. Study Flow Diagram eReferences. eTable 1. Characteristics of the Randomized Clinical Trials in Our Systematic Review eTable 2. Data Synthesis eTable 3. Transitivity Assessment eTable 4. Risk of Bias Summary eAppendix 1. Negative Symptoms eAppendix 2. Overall Symptoms eAppendix 3. Positive Symptoms eAppendix 4. PANSS General Subscale Score eAppendix 5. Depressive Symptoms eAppendix 6. Anxiety Symptoms eAppendix 7. Overall Cognitive Impairment eAppendix 8. All-Cause Discontinuation eAppendix 9. Discontinuation Due to Adverse Events eAppendix 10. Headache eAppendix 11. Dizziness [file jamanetwopen-e2441159-s001.pdf]

Supplemental Online Content

Kishi T, Ikuta T, Sakuma K, et al. Comparative efficacy and acceptability of theta burst stimulation protocols for schizophrenia: a systematic review and network meta-analysis. *JAMA Netw Open.* 2024;7(10):e2441159. doi:10.1001/jamanetworkopen.2024.41159

eFigure. Study Flow Diagram

eReferences.

eTable 1. Characteristics of the Randomized Clinical Trials in Our Systematic Review

eTable 2. Data Synthesis

eTable 3. Transitivity Assessment

eTable 4. Risk of Bias Summary

eAppendix 1. Negative Symptoms

eAppendix 2. Overall Symptoms

eAppendix 3. Positive Symptoms

eAppendix 4. PANSS General Subscale Score

eAppendix 5. Depressive Symptoms

eAppendix 6. Anxiety Symptoms

eAppendix 7. Overall Cognitive Impairment

eAppendix 8. All-Cause Discontinuation

eAppendix 9. Discontinuation Due to Adverse Events

eAppendix 10. Headache

eAppendix 11. Dizziness

This supplemental material has been provided by the authors to give readers additional information about their work.

© 2024 Kishi T et al. *JAMA Network Open.*

eFigure. Study Flow Diagram

We searched the PubMed, the Cochrane Library, and Embase databases for studies published before May 22, 2024, without language restriction. The search terms for PubMed and the Cochrane Library included (theta burst stimulation OR TBS OR iTBS OR cTBS) AND (random*) AND (schizophreni* OR psychosis OR psychotic). The search terms for Embase included ('theta burst stimulation'/exp OR 'theta burst stimulation') AND ('psychosis'/exp OR psychosis) AND ('randomized controlled trial'/exp OR 'randomized controlled trial'). Additionally, reference lists of the included articles were manually searched for additional relevant published and unpublished research, including conference abstracts. We also searched clinical trial registries (ClinicalTrials.gov [http://clinicaltrials.gov/] and the World Health Organization International Clinical Trials Registry Platform [http://www.who.int/ictrp/search/en/]) to ensure the RCTs were comprehensive and to minimize the effect of publication bias. Any discrepancies in the selected articles were resolved by consensus of the authors. If multiple papers or academic conference abstracts were reported despite the same research, the literature was screened by confirming the clinical trial registration number and/or reference to past review articles.

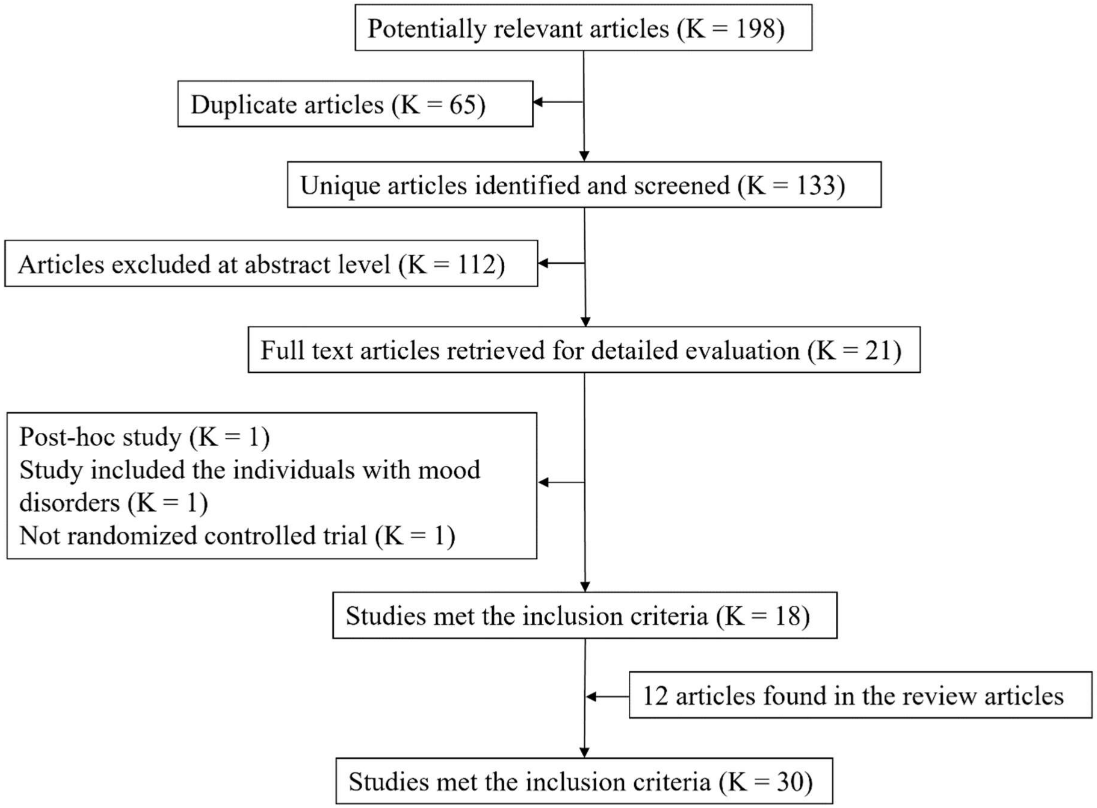

© 2024 Kishi T et al. *JAMA Network Open.*

eReferences.

Review articles that we read for the literature search

1. Dougall N, Maayan N, Soares-Weiser K, McDermott LM, McIntosh A. Transcranial magnetic stimulation (TMS) for schizophrenia. Cochrane Database Syst Rev 2015; 2015(8): CD006081.

2. Goh KK, Chen CH, Wu TH, Chiu YH, Lu ML. Efficacy and safety of intermittent theta-burst stimulation in patients with schizophrenia: A meta-analysis of randomized sham-controlled trials. Front Pharmacol 2022; 13: 944437.

3. Hyde J, Carr H, Kelley N, Seneviratne R, Reed C, Parlatini V et al. Efficacy of neurostimulation across mental disorders: systematic review and meta-analysis of 208 randomized controlled trials. Mol Psychiatry 2022; 27(6): 2709-2719.

4. Lorentzen R, Nguyen TD, McGirr A, Hieronymus F, Ostergaard SD. The efficacy of transcranial magnetic stimulation (TMS) for negative symptoms in schizophrenia: a systematic review and meta-analysis. Schizophrenia (Heidelb) 2022; 8(1): 35.

5. Marzouk T, Winkelbeiner S, Azizi H, Malhotra AK, Homan P. Transcranial Magnetic Stimulation for Positive Symptoms in Schizophrenia: A Systematic Review. Neuropsychobiology 2020; 79(6): 384-396.

6. Poorganji M, Goeke K, Zomorrodi R, Voineskos D, Rajji TK, Daskalakis ZJ et al. The use of theta burst stimulation in patients with schizophrenia - A systematic review. Schizophr Res 2023; 261: 245-255.

7. Salabat D, Pourebrahimi A, Mayeli M, Cattarinussi G. The Therapeutic Role of Intermittent Theta Burst Stimulation in Schizophrenia: A Systematic Review and Meta-analysis. J ECT 2024.

8. Tan X, Goh SE, Lee JJ, Vanniasingham SD, Brunelin J, Lee J et al. Efficacy of Using Intermittent Theta Burst Stimulation to Treat Negative Symptoms in Patients with Schizophrenia-A Systematic Review and Meta-Analysis. Brain Sci 2023; 14(1).

9. Tseng PT, Zeng BS, Hung CM, Liang CS, Stubbs B, Carvalho AF et al. Assessment of Noninvasive Brain Stimulation Interventions for Negative Symptoms of Schizophrenia: A Systematic Review and Network Meta-analysis. JAMA Psychiatry 2022; 79(8): 770-779.

10. Wang J, Zhou Y, Gan H, Pang J, Li H, Wang J et al. Efficacy Towards Negative Symptoms and Safety of Repetitive Transcranial Magnetic Stimulation Treatment for Patients with Schizophrenia: A Systematic Review. Shanghai Arch Psychiatry 2017; 29(2): 61-76.

11. Zhang X, Yang X, Shi Z, Xu R, Tan J, Yang J et al. A Systematic Review of Intermittent Theta Burst Stimulation for Neurocognitive Dysfunction in Older Adults with Schizophrenia. J Pers Med 2023; 13(3).

© 2024 Kishi T et al. *JAMA Network Open.*

eTable 1. Characteristics of the Randomized Clinical Trials in Our Systematic Review

Study name (registry)	Design (duration)	Sponsorship	Country	Diagnosis (status)	Minimum of symptoms at baseline	Total n	BL scores (mean±SD)	%Female	Age (mean±SD)	Treatment arms	Coil	Device	Locating	TBS treatment d	MT (%)	Frequency (Hz)	Total pulses/session	Total session/d	Total session	Total pulses	Intersession interval	AP information
Basavaraju 2021 (CTRI/2017/09/009636)	DBRSCT (6 w)	Academia	India	SZ (DSM-5, OP and OP)	SANS each item≥3	60	SAPS: 5.37±7.87, SANS: 83.85±12.79	23.33	32.67±9.08	iTBS (CV)	F8	MagProX 100	MRI	5	100 (AMT)	50	600	2	10	6000	4 h	CLO (18.33%), AP-POL (25.00%)
										Sham coil												
Bation 2021 (NCT00875498)	DBRSCT (6 m)	Academia	France	SZ (DSM-IV-TR, OP)	PANSS-N≥20 (≥2 items≥4)	22	PANSS-T: 75.50±11.76, PANSS-P: 10.14±2.55, SANS: 74.73±17.10	4.55	42.0±10.73	iTBS (L-DLPFC)	F8	MagProX 100	6 cm rule	10	80 (RMT)	50	990	2	20	19800	≥2 h	CHL eq: 354.14±189.31 mg/d
										Sham coil												
Brady 2019 (NCT01561859)	DBRSCT (1 w)	Academia	USA	SZ or SA (DSM-IV-TR, IP and OP)	No	17	PANSS-T: 75.91±22.32, PANSS-P: 17.09±5.82, PANSS-N: 23.00±10.65	27.27	35.55±10.50	iTBS (CV)	F8	MagPro X100	MRI	5	100 (AMT)	50	600	2	10	6000	≥4 h	CHL eq: 614.20±606.50 mg/d
										Sham coil												
Chauhan 2021 (ISRCTN49569866)	DBRSCT (3 w)	Academia	India	TRSZ (ICD-10, Kane's criteria, IP)	BPRS-T>45 (≥2 positive items>4)	36	PANSS-T: 91.97±8.79, PANSS-P: 20.91±2.38, PANSS-N: 26.95±3.22	58.33	40.61±8.52	iTBS (CV)	F8	MagProR30	EEG	5	80 (RMT)	50	600	2	10	6000	≥30 min	CHL eq: 588.89±133.69 mg/d
										Sham coil												
Chen 2011	DBRSCT (4 w)	Academia	China	SZ (DSM-IV, IP)	PANSS-N≥20	46	PANSS-T: 74.76±6.10	35.71	38.44±12.40	iTBS (L-DLPFC)	Circular	MagProX 100	EEG	20	80 (RMT)	50	2400	1	20	48000	na	FGAs and SGAs, CHL eq: 487.36±143.11 mg/d
										Sham coil												
Gan 2014	DBRSCT (NR)	NR	China	SZ (NR)	NR	41	PANSS-N: 22.59±3.32	NR	26.51±7.43	iTBS (L-DLPFC)	NR	NR	EEG	NR	100 (NR)	50	NR	NR	NR	NR	NR	NR
										Sham coil												
Jin 2021	DBRSCT (4 w)	Academia	China	SZ (ICD-10, IP)	SANS≥35	80	PANSS-T: 66.10±6.40, PANSS-P: 12.55±3.17, PANSS-N: 23.05±3.56	56.25	48.20±10.10	iTBS (L-DLPFC)	Circular	JunJiang RT-100	NR	20	100 (RMT)	50	NR	1	20	NR	na	SGA (100%), CHL eq: 262.34±61.16 mg/d
										180° coil angulation												
Jin 2023 (ChiCTR2100051984)	DBRSCT (4 w)	Academia	China	SZ (DSM-5, NR)	No	66	PANSS-T: 66.00±5.05, PANSS-P: 12.74±2.69, PANSS-N: 23.52±3.17	43.94	47.53±10.17	iTBS (L-DLPFC)	F8	Brain Ultimate M-100	MRI	20	120 (RMT)	50	600	3	60	36000	15 min	OLA (100%): 11.98±2.69 mg/d
										Sham coil												
Kang 2023 (NCT05358899)	DBRSCT (40 d)	Academia	China	SZ (DSM-5, IP) with BMI>24	No	40	PANSS-T: 68.72±15.40, PANSS-P: 14.80±5.67, PANSS-N: 22.09±6.16	70.00	29.32±8.52	cTBS (L-M1)	NR	NR	Contraction of R-thumb	10	80 (RMT)	50	1800	5	50	90000	60 min	CHL eq: 340.65±225.75 mg/d
										90° coil angulation												
Kang 2024 (NCT05086133)	DBRSCT (5 d)	Academia	China	FEDNSZ (DSM-5, IP)	No	39	PANSS-T: 74.85±12.00, PANSS-P: 18.39±4.71,	86.11	25.65±7.35	cTBS (L-M1)	NR	NR	Contraction of R-thumb	5	80 (RMT)	50	600	5	25	15000	60 min	OLA (100%): 14.25±4.40 mg/d

© 2024 Kishi T et al. *JAMA Network Open.*

							PANSS-N: 21.14±6.16															
										90° coil angulation												
Kazemi 2012	DBRSCT (4 w)	Academia	Iran	SZ (DSM-IV-TR, NR)	PANSS-P≥15 and PANSS-N≥15	10	PANSS-T: 114.60±16.11	NR	27.10±3.56	iTBS (L-DLPFC)	F8	Magstim Rapid 2	5 cm rule	20	80 (RMT)	50	600	1	20	12000	na	NR
										Sham coil												
Koops 2016 (NCT01512290)	DBRSCT (1 m)	Academia	Netherlands	SZ (67.61%), SA, or SFD (DSM-IV-TR, NR)	No	71	PANSS-T: 71.56±19.91	43.66	39.92±14.12	cTBS (L-TPC) (between T3 and P3)	F8	Magstim Rapid 2	EEG	5	80 (RMT)	50	900	2	10	9000	30 min	CLO (45.07%), none (8.45%)
										Sham coil												
Kos 2024 (NTR3805)	DBRSCT (10 w)	Academia	Netherlands	SZ or SA (DSM-IV, IP and OP)	AES-C≥27	48	PANSS-T: 64.10±14.47, PANSS-N: 18.23±4.03	25.00	33.00-34.00*	iTBS (R-DLPFC)	F8	MagPro X100 or R30	EEG	10	80 (RMT)	30	990	1	10	9900	na	NR
										Sham coil												
Mao 2019	DBRSCT (4 w)	NR	China	SZ (NR, IP)	PANSS-N≥18	60	NR	NR	53.15±6.31	iTBS (L-DLPFC)	NR	MagPro R100	NR	20	80 (NR)	50	NR	1	20	NR	na	NR
										NR												
Sun 2017	DBRSCT (8 w)	NR	China	SZ (ICD-10, IP)	PANSS-N≥20	100	NR	NR	51.05±11.70	iTBS (L-DLPFC)	NR	NR	NR	40	80 (NR)	50	2400	1	40	96000	na	NR
										No treatment												
Tikka 2017 (CTRI-2014-12-005280)	DBRSCT (2 w)	Academia	India	SZ (ICD-10, IP)	No (FRS)	20	PANSS-T: 71.87±12.98, PANSS-P: 24.80±3.47, PANSS-N: 17.33±5.34	0.00	26.95±4.25	cTBS (R-IPL)	F8	Magstim Rapid 2	MRI	10	80 (RMT)	50	900	1	10	9000	na	AP (100%), CHL eq: 330.63±66.49 mg/d
										Sham coil												
Tyagi 2022 (CTRI/2020/12/029506)	DBRSCT (2 w)	Academia	India	SZ (ICD-10, IP)	PANSS((P3)≥3	59	PANSS-T: 90.41±11.31, PANSS-P: 27.95±4.06, PANSS-N: 29.15±5.38	35.59	32.73±11.27	cTBS (L and R-TPC) (between T3/P3 and T4/P4)	F8	MagPro R30	EEG	10	80 (RMT)	50	600(L)/600(R)	2	20	12000(L)/12000(R)	immediately	CHL eq: 644.91±47.06 mg/d
										Sham coil												
Vergallito 2024	DBRSCCT (6 m)	Academia	Italy	SZ (70.00%), SA or PDNOS (DSM-5, OP)	No	10	PANSS-T: 64.91±19.15	40.00	36.5±10.36	iTBS (L-DLPFC)	F8	Magstim Rapid2	EEG	15	100 (AMT)	50	600	1	15	9000	na	NR
										Sham coil												
Walther 2020 (NCT03483909)**	DBRSCCT (3 d)	Academia	Switzerland	SZ (DSM-5, IP and OP)	No	20	PANSS-T: 96.6±24.2, PANSS-P: 21.7±10.5, PANSS-N: 28.4±7.4	40.00	34.3±12.6	cTBS (R-IPL) (CP4/6)	F8	MagPro R30	EEG	1	100 (RMT)	30	801	1	1	801	na	CHL eq: 478.9±638.1 mg/d
										iTBS (L-IFG) (between F5/F7/FC5/FT7)	F8	MagPro R30	EEG	1	80 (RMT)	50	600	1	1	600	na	
										Sham coil												
Walther 2024 (NCT03921450)	DBRSCT (24 w)	Academia	Switzerland	SZ (DSM-5, IP)	SRRS≥15	53	PANSS-T: 79.55±17.20, PANSS-P: 15.95±5.44, PANSS-N: 23.95±6.43	52.27	35.85±11.96	iTBS (L-SMA)	F8	MagPro X100 or R30	3 cm rule	15	80 (RMT)	50	600	2	30	18000	10 min	OLA eq: 18.00±12.56 mg/d
										Sham coil												
Wang 2020	DBRSCT (8 w)	Academia	China	SZ (DSM-IV, OP)	No	58	PANSS-T: 63.90±15.27, PANSS-P:	56.00	25.32±7.16	iTBS (L-DLPFC)	F8	Magstim Rapid2	MRI	14	80 (RMT)	50	600	3	42	25200	15 min	AP (100%),

© 2024 Kishi T et al. *JAMA Network Open.*

(NCT03 868358)							14.10±5.48, PANSS-N: 16.22±4.40															OLA eq: 20.96±16.00 mg/d
										Sham coil												
Wang 2022	DBRSCT (2 w)	Academia	China	TRSZ (DSM-IV, OP)	No	59	PANSS-T: 71.25±18.63, PANSS-P: 15.44±6.71, PANSS-N: 17.51±4.65	55.93	23.95±4.99	iTBS (L-DLPFC)	F8	Magstim Rapid 2	MRI	14	80 (RMT)	50	600	3	42	25200	15 min	SGA (100%), OLA eq: 18.51±14.22 mg/d
										Sham coil												
Wu 2021 (NCT03 868358)	DBRSCT (2 w)	Academia	China	SZ (DSM-IV, OP)	No	36	PANSS-T: 59.60±12.06, PANSS-P: 13.06±4.55, PANSS-N: 15.69±4.14	NR	24.06±7.41	iTBS (L-DLPFC)	F8	Magstim Rapid 2	MRI	14	80 (RMT)	50	600	3	42	25200	15 min	AP (100%), OLA eq: 4.87±1.39 mg/d
										Sham coil												
Zhang 2010	DBRSCT (4 w)	NR	China	SZ (DSM-IV, NR)	NR	30	PANSS-N: 26.3±3.01	36.67	27.50±7.88	iTBS (L-DLPFC)	NR	NR	NR	20	80 (NR)	50	2400	1	20	48000	na	NR
										Sham coil												
Zhao 2014	DBRSCT (4 w)	Academia	China	SZ (DSM-IV, IP and OP)	PANSS-N≥20 (≥1 items≥4)	48	PANSS-T: 77.15±8.12, PANSS-P: 14.16±2.75, PANSS-N: 38.65±5.48	45.65	47.22±12.31	iTBS (L-DLPFC)	Circular	MagPro R100	NR	20	80 (NR)	50	2400	1	20	48000	na	NR
										180° coil angulation												
Zhao 2021	DBRSCT (4 w)	NR	China	SZ (ICD-10, IP)	NR	52	PANSS-T: 60.60±3.27	33.30	63.25±3.50	iTBS (L-DLPFC)	NR	NR	NR	20	100 (NR)	50	600	1	20	12000	na	CHL eq: 511.00 mg/d
										90° coil angulation												
Zhen 2015	DBRSCT (4 w)	Academia	China	SZ (DSM-IV, IP)	NR	60	NR	56.14	43.44±12.48	iTBS (L-DLPFC)	F8	Magpro R100	NR	20	80 (NR)	50	1200	1	20	24000	na	NR
										180° coil angulation												
Zhen 2018	DBRSCT (4 w)	NR	China	SZ (DSM-IV, IP)	PANSS each item<5	80	NR	54.55	63.43±12.50	iTBS (L-DLPFC)	F8	Magpro R100	NR	20	80 (NR)	50	1200	1	20	24000	na	HAL eq: 9.85±2.61 mg/d
										180° coil angulation												
Zheng 2012	DBRSCT (5 d)	Academia	China	SZ (CCMD-3, IP)	No	39	PANSS-T: 66.03±12.21. PANSS-P: 12.56±4.80, PANSS-N: 23.21±5.19	0.00	56.01±7.70	iTBS (L-DLPFC)	Circular	Magpro X100	NR	5	80 (NR)	50	1200	1	5	6000	na	CLO (65.71%)
										180° coil angulation												
Zhu 2021 (NCT03 940898)	DBRSCT (24 w)	Academia	China	SZ (ICD-10, IP)	PANSS-P1/P3/P5/P6/G9≤5, P2≤4	64	PANSS-T: 65.36±19.28, PANSS-P: 13.75±5.31, PANSS-N: 18.75±8.04	50.00	35.25±6.60	iTBS (CV)	F8	Magpro X100 or CCY-1	EEG	10	100 (RMT)	50	600	1	10	6000	na	AP (100%), CHL eq: 475.63±260.67 mg/d
										90° or 180° coil angulation												

*The median age in each treatment group.

**The study was not included in the meta-analysis because the study duration was short.

AES-C: Apathy Evaluation Scale Clinical version, AMT: active motor threshold, AP: antipsychotic(s), AP-POL: antipsychotic polypharmacy, BMI: body mass index, BPRS-T: Brief Psychiatric Rating Scale total score, CCMD: Chinese classification of mental disorders, CHL: chlorpromazine, CLO: clozapine, cTBS: continuous theta burst stimulation, CV: cerebellar

© 2024 Kishi T et al. *JAMA Network Open.*

vermis, DBRSCT: double-blind randomized sham-controlled trial, DBRSCCT: double-blind randomized sham-controlled, crossover trial, d: day(s), DSM (-TR): Diagnostic and Statistical Manual of Mental Disorders (-Text Revision), EEG: electroencephalography, eq: equivalent dose, F8: Figure-of-eight coil, FEDNSZ: first-episode drug-naïve schizophrenia, FGA: first generation antipsychotic(s), FRS: first-rank symptoms, h: hours, HAL: haloperidol, Hz: hertz, ICD: International Statistical Classification of Diseases and Related Health Problems, IP: inpatient, iTBS: intermittent theta burst stimulation, L (or R-) DLPFC: (left-, right-) dorsolateral prefrontal cortex, L-IFG: left inferior frontal gyrus, L-M1:left- primary motor cortex, L-SMA: left supplementary motor are, L (or R-) TPC: (left-, right-) temporoparietal cortex, m: month, min: minute(s), MRI: magnetic resonance imaging, MT: motor threshold, n: number of individuals, na: not applicable, NR: not report, OLA: olanzapine, OP: outpatient, PANSS (-T, -P, -N): Positive and Negative Syndrome Scale (-total score, -positive subscale score, -negative subscale score), PDNOS: psychotic disorder not otherwise specified, R-IPL: right inferior parietal lobule, RMT: resting motor threshold, SA: schizoaffective disorder, SANS: Scale for the Assessment of Negative Symptoms, SAPS: Scale for the Assessment of Positive Symptoms, SD: standard deviation, SFD: schizophreniform disorder, SGA: second generation antipsychotic(s), SRRS: Salpêtrière Retardation Rating Scale, SZ: schizophrenia, TBS: theta burst stimulation, TRSZ: treatment-resistant schizophrenia, USA: United States of America, w: weeks

© 2024 Kishi T et al. *JAMA Network Open.*

eTable 2. Data Synthesis

	Outcomes*												
Study name	PANSS-T[1]	PANSS-P[1]	SAPS[2]	PANSS-N[1]	SANS[3]	PANSS-G[1]	CDSS[4]	HAMD[5]	AES[6]	HAMA[7]	SCoRS[8]	MoCA[9]	MCCB[10,11]
Basavaraju 2021			✓		✓		✓						
Bation 2021		✓		✓		✓							
Brady 2019	✓	✓		✓		✓							
Chauhan 2021	✓	✓		✓		✓					✓		
Chen 2011	✓	✓		✓		✓							
Gan 2014				✓									
Jin 2021		✓		✓									
Jin 2023	✓	✓		✓		✓							
Kang 2023	✓	✓		✓		✓		✓		✓			
Kang 2024	✓	✓		✓		✓							
Kazemi 2012	✓	✓		✓									
Koops 2016		✓											
Kos 2024				✓	✓				✓				
Mao 2019		✓		✓									
Sun 2017	✓				✓								
Tikka 2017	✓	✓		✓		✓							
Tyagi 2022	✓	✓		✓		✓	✓				✓		
Vergallito 2024	✓	✓		✓		✓	✓						✓
Walther 2024	✓	✓		✓		✓							
Wang 2020	✓	✓	✓	✓	✓	✓		✓		✓		✓	
Wang 2022	✓	✓	✓	✓	✓	✓		✓		✓		✓	
Wu 2021	✓	✓	✓	✓	✓	✓		✓		✓			
Zhang 2010	✓	✓		✓	✓	✓							
Zhao 2014	✓			✓	✓	✓							

© 2024 Kishi T et al. *JAMA Network Open.*

Zhao 2021	✓	✓		✓									
Zhen 2015													
Zhen 2018													
Zheng 2012	✓	✓		✓		✓							
Zhu 2021	✓	✓		✓		✓							

*Studies for which data were used in the meta-analysis of each outcome were marked with "✓."

1. Kay SR, Fiszbein A, Opler LA. The positive and negative syndrome scale (PANSS) for schizophrenia. Schizophr Bull. 1987;13(2):261-276.
2. Andreasen N. Scale for the assessment of positive symptoms (SAPS). Univeristy of Iowa. 1984.
3. Andreasen NC. Negative symptoms in schizophrenia. Definition and reliability. Arch Gen Psychiatry. 1982;39(7):784-788.
4. Addington D, Addington J, Schissel B. A depression rating scale for schizophrenics. Schizophr Res. 1990;3(4):247-251.
5. Hamilton M. Development of a rating scale for primary depressive illness. Br J Soc Clin Psychol. 1967;6(4):278-296.
6. Faerden A, Lyngstad SH, Simonsen C, et al. Reliability and validity of the self-report version of the apathy evaluation scale in first-episode Psychosis: Concordance with the clinical version at baseline and 12 months follow-up. Psychiatry Res. 2018;267:140-147.
7. Hamilton M. The assessment of anxiety states by rating. Br J Med Psychol. 1959;32(1):50-55.
8. Keefe RS, Poe M, Walker TM, Kang JW, Harvey PD. The Schizophrenia Cognition Rating Scale: an interview-based assessment and its relationship to cognition, real-world functioning, and functional capacity. Am J Psychiatry. 2006;163(3):426-432.
9. Nasreddine ZS, Phillips NA, Bedirian V, et al. The Montreal Cognitive Assessment, MoCA: a brief screening tool for mild cognitive impairment. J Am Geriatr Soc. 2005;53(4):695-699.
10. Kern RS, Nuechterlein KH, Green MF, et al. The MATRICS Consensus Cognitive Battery, part 2: co-norming and standardization. Am J Psychiatry. 2008;165(2):214-220.
11. Nuechterlein KH, Green MF, Kern RS, et al. The MATRICS Consensus Cognitive Battery, part 1: test selection, reliability, and validity. Am J Psychiatry. 2008;165(2):203-213.

© 2024 Kishi T et al. *JAMA Network Open.*

eTable 3. Transitivity Assessment

	Boxplot	Kruskal–Wallis equality of populations rank test for continuous variables or the Pearson chi-square test for binary and categorical variables (or the Fisher's exact test whether >20% of cells had an expected frequency below 5).*
Individuals with predominantly negative symptoms (k = 29)	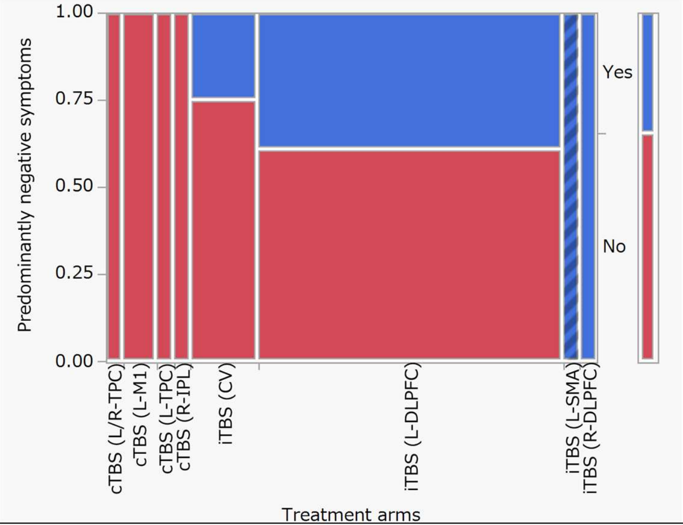	Chi-squared with ties = 6.745 (df = 7), p = 0.456
Proportion of females (k = 24)	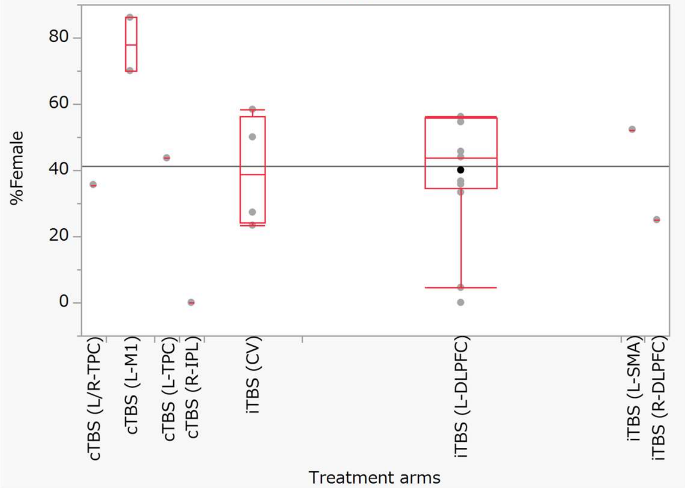	Chi-squared with ties = 9.091 (df = 7), p = 0.246

© 2024 Kishi T et al. *JAMA Network Open.*

Mean age (k = 29)	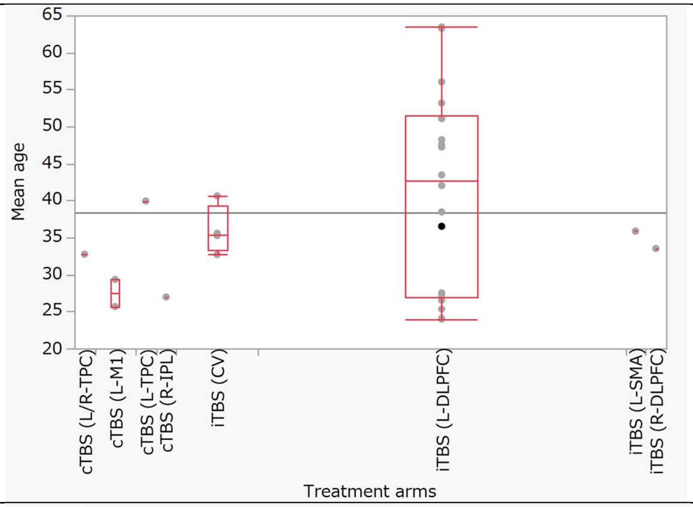	Chi-squared with ties = 4.520 (df = 7), p = 0.718
Total number of participants (k = 29)	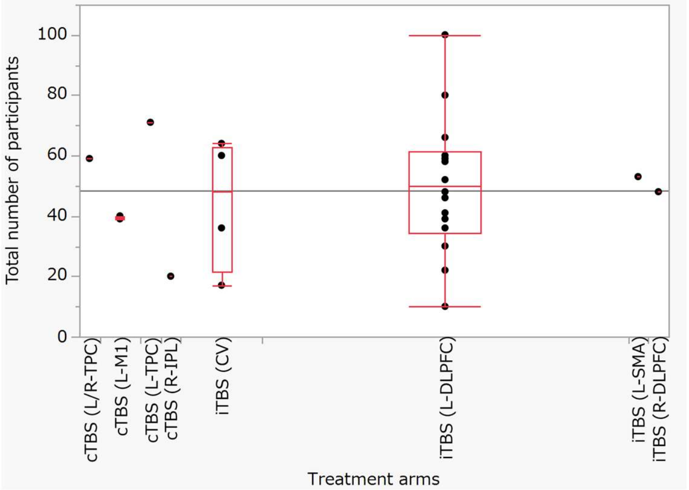	Chi-squared with ties = 7.987 (df = 7), p = 0.334

© 2024 Kishi T et al. *JAMA Network Open.*

Antipsychotic dose (k = 17)	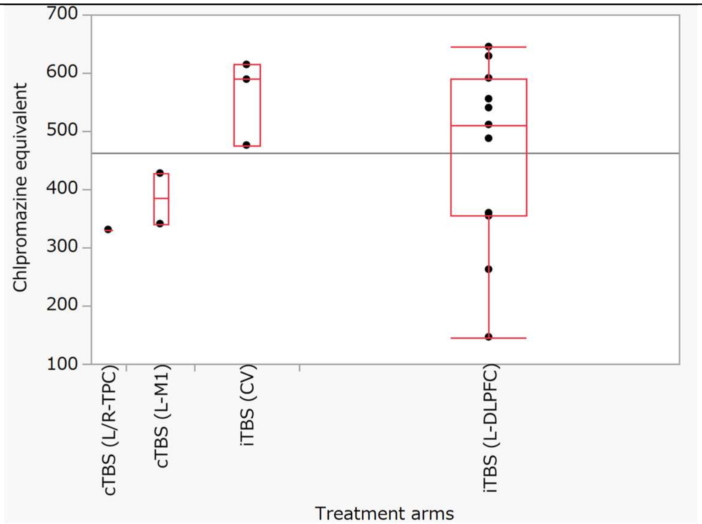	Chi-squared with ties = 3.488 (df = 3), p = 0.322
Coil localization/targeting method (k = 20)	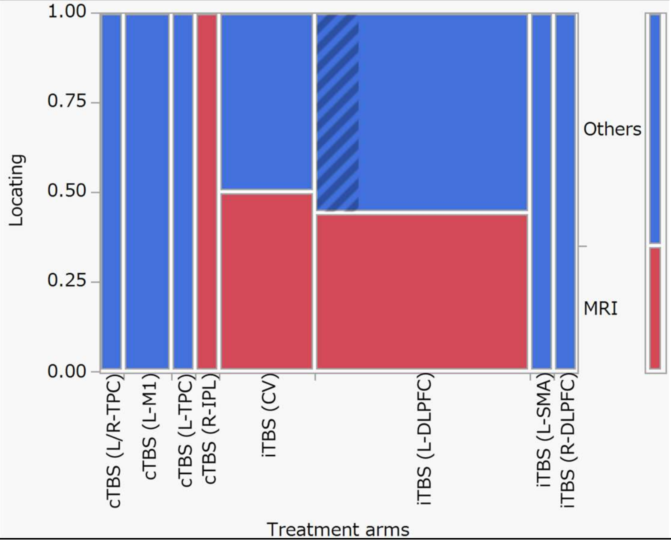	Chi-squared with ties = 4.389 (df = 7), p = 0.734

© 2024 Kishi T et al. *JAMA Network Open.*

TBS coil (k = 22)	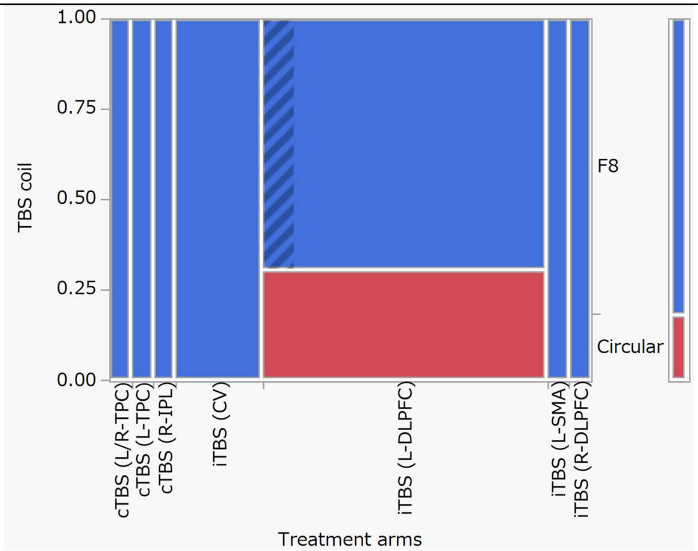	Chi-squared with ties = 3.385 (df = 6), p = 0.759
Use of sham coil (k = 28)	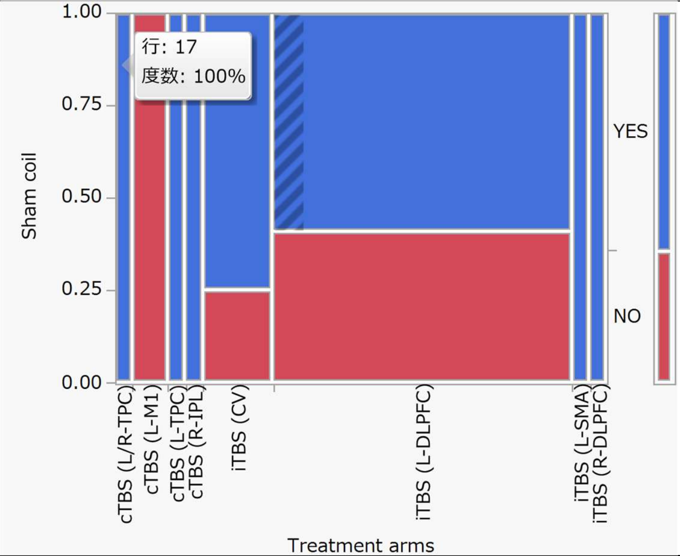	Chi-squared with ties = 6.799 (df = 7), p = 0.450

© 2024 Kishi T et al. *JAMA Network Open.*

Percent motor threshold (k = 29)	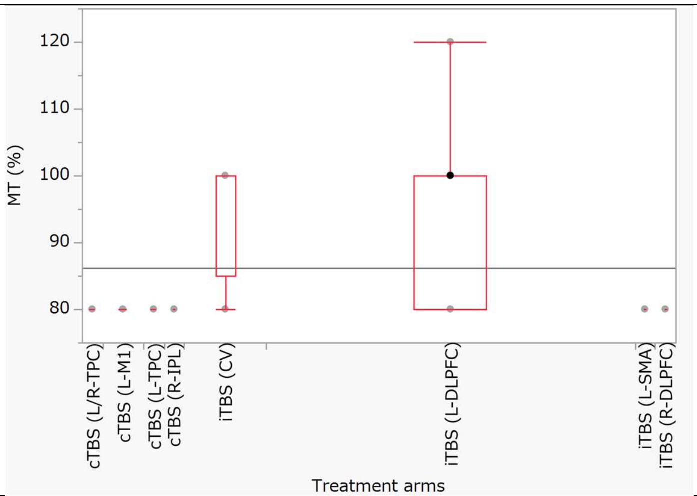	Chi-squared with ties = 6.391 (df = 7), p = 0.494
Number of sessions during a day (k = 28)	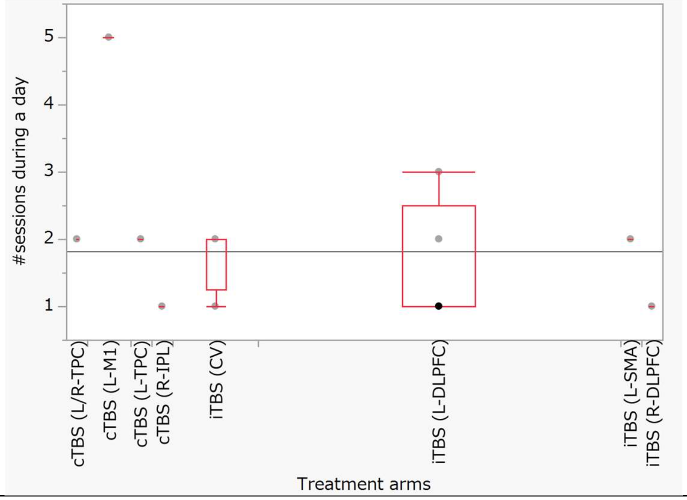	Chi-squared with ties = 10.017 (df = 7), p = 0.188

© 2024 Kishi T et al. *JAMA Network Open*.

Number of sessions during a trial (k = 29)	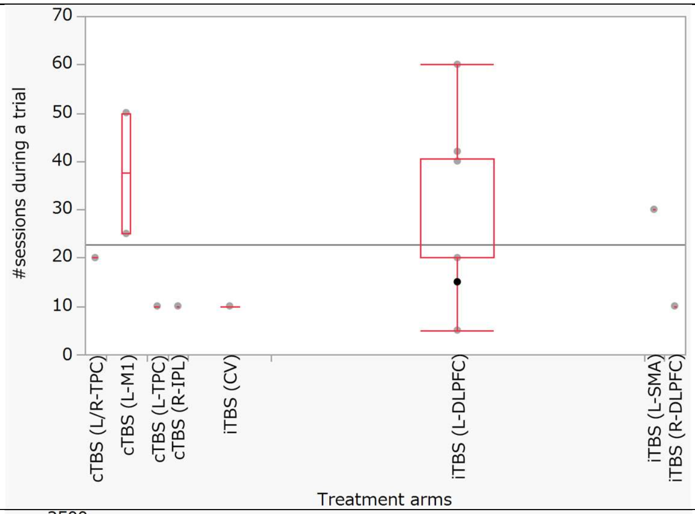	Chi-squared with ties = 15.982 (df = 7), p = 0.0253
Number of pulses during a session (k = 26)	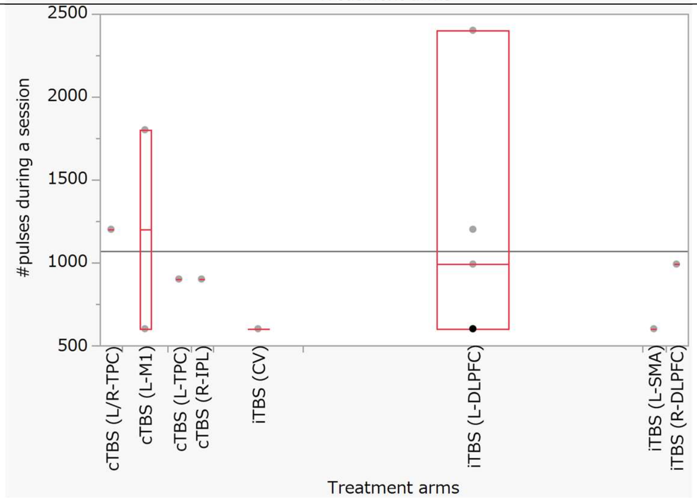	Chi-squared with ties = 5.624 (df = 7), p = 0.584

© 2024 Kishi T et al. *JAMA Network Open.*

Number of pulses during a trial (k = 26)	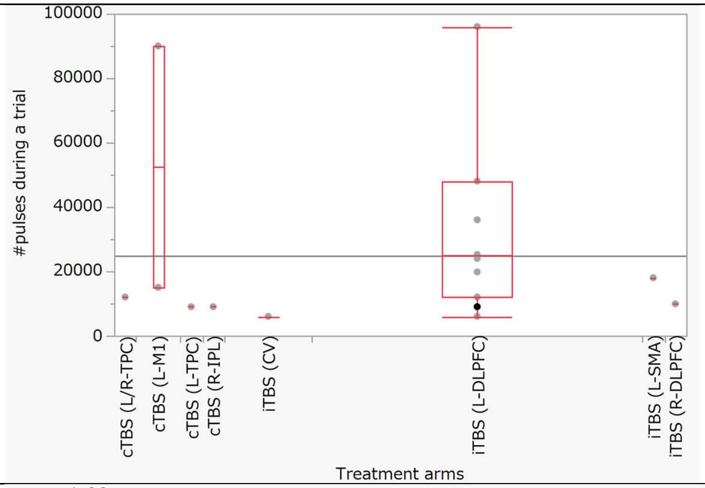	Chi-squared with ties = 13.552 (df = 7), p = 0.060
Negative symptoms scales (k = 26)	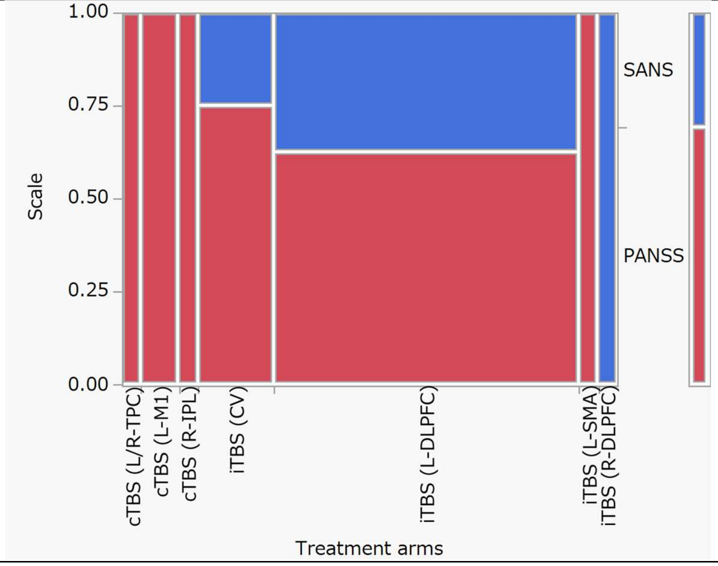	Chi-squared with ties = 4.875 (df = 6), p = 0.560

© 2024 Kishi T et al. *JAMA Network Open.*

Publication year (k = 29)	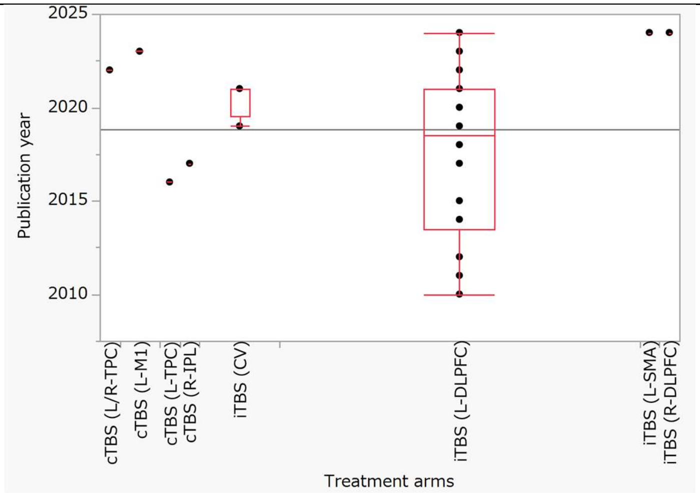	Chi-squared with ties = 11.357 (df = 7), p = 0.124
Country where the trial was conducted (k = 29)	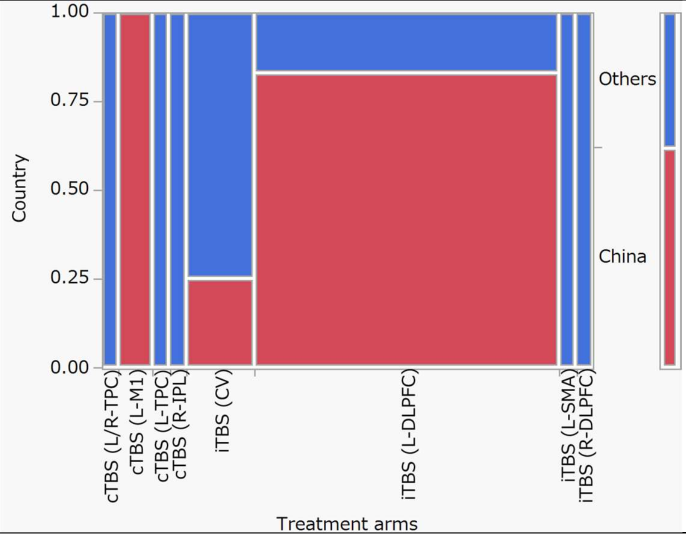	Chi-squared with ties = 15.196 (df = 7), p = 0.0336

© 2024 Kishi T et al. *JAMA Network Open.*

Overall risk of bias (k = 29)	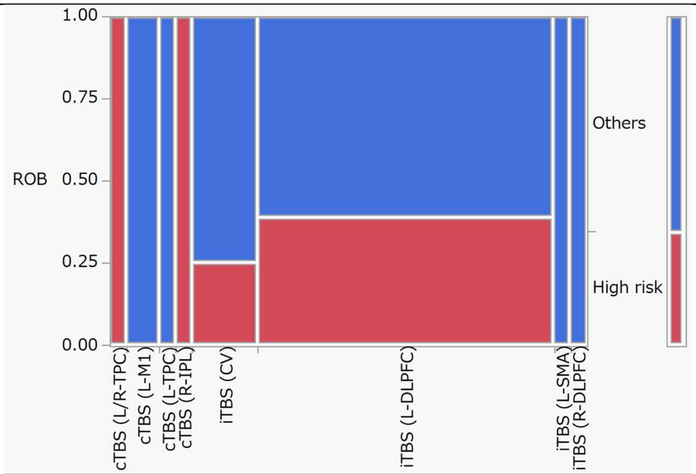	Chi-squared with ties = 8.807 (df = 7), p = 0.267

*When the *p*-value was <0.1, we considered that there was a difference in the data among the TBS protocols.

© 2024 Kishi T et al. *JAMA Network Open.*

eTable 4. Risk of Bias Summary

	Randomization process	Deviation from intended intervention	Missing outcome data	Measurement of the outcome	Selection of the reported result	Overall risk of bias
Basavaraju 2021	Low risk	Low risk	Low risk	Low risk	Some concerns	Some concerns
Bation 2021	Some concerns	Low risk	Some concerns	Low risk	Low risk	Some concerns
Brady 2019	Some concerns	High risk	High risk	Low risk	Some concerns	High risk
Chauhan 2021	Low risk	Low risk	Low risk	Low risk	Low risk	Low risk
Chen 2011	Some concerns	Some concerns	Low risk	Some concerns	Low risk	Some concerns
Gan 2014	Some concerns	Some concerns	Some concerns	Some concerns	Some concerns	Some concerns
Jin 2021	Some concerns	Some concerns	High risk	Low risk	Some concerns	High risk
Jin 2023	Some concerns	Low risk	Low risk	Low risk	Some concerns	Some concerns
Kang 2023	Low risk	Low risk	Some concerns	Low risk	Some concerns	Some concerns
Kang 2024	Low risk	Low risk	Low risk	Low risk	Some concerns	Some concerns
Kazemi 2012	Some concerns	Low risk	Low risk	Some concerns	Some concerns	Some concerns
Koops 2016	Low risk	Low risk	Low risk	Low risk	Some concerns	Some concerns
Kos 2024	Low risk	Some concerns	Low risk	Low risk	Some concerns	Some concerns
Mao 2019	Some concerns	Some concerns	Some concerns	Some concerns	Some concerns	Some concerns
Sun 2017	Some concerns	Some concerns	Some concerns	Some concerns	Some concerns	Some concerns
Tikka 2017	Low risk	High risk	High risk	Low risk	Some concerns	High risk
Tyagi 2022	High risk	Low risk	Low risk	Low risk	Some concerns	High risk
Vergallito 2024	Some concerns	Low risk	Low risk	High risk	Some concerns	High risk
Walther 2024	Low risk	Low risk	Some concerns	Low risk	Low risk	Low risk
Wang 2020	Some concerns	High risk	High risk	Low risk	Some concerns	High risk
Wang 2022	Some concerns	Low risk	Some concerns	Some concerns	Some concerns	Some concerns
Wu 2021	Some concerns	High risk	High risk	Low risk	Some concerns	High risk
Zhang 2010	Some concerns	Low risk	Low risk	High risk	High risk	High risk
Zhao 2014	Some concerns	Low risk	Low risk	Low risk	Low risk	Some concerns
Zhao 2021	Some concerns	Some concerns	Some concerns	Some concerns	Some concerns	Some concerns
Zhen 2015	Low risk	High risk	High risk	Some concerns	Some concerns	High risk
Zhen 2018	Some concerns	High risk	Low risk	High risk	Some concerns	High risk
Zheng 2012	Some concerns	Low risk	Low risk	Low risk	Low risk	Some concerns
Zhu 2021	Some concerns	Low risk	Low risk	Low risk	Low risk	Some concerns

Version 2 of the Cochrane risk-of-bias tool for randomized trials (https://www.riskofbias.info/)

© 2024 Kishi T et al. *JAMA Network Open.*

eAppendix 1. Negative Symptoms

Network meta-analysis

26 studies, 1124 participants

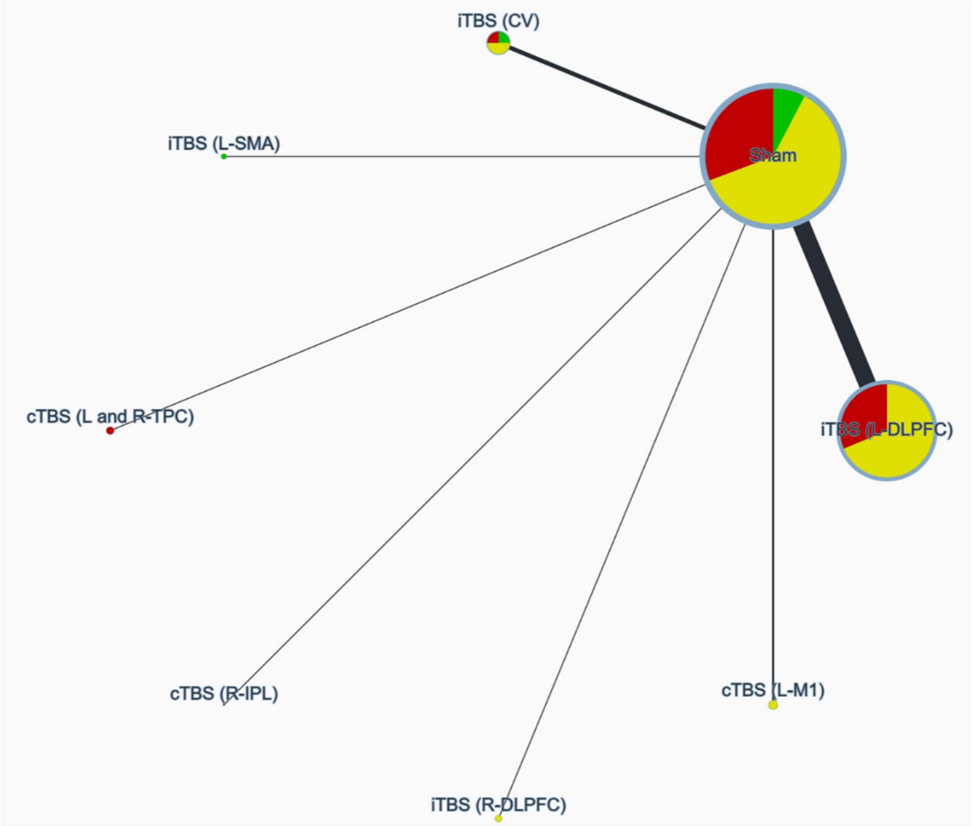

Node size by sample size

Node color by risk of bias

Green: low overall risk of bias

Yellow: moderate overall risk of bias

Red: high overall risk of bias

Edge width by number of studies

© 2024 Kishi T et al. *JAMA Network Open.*

League table (SMD with 95% confidence interval)

cTBS (L and R-TPC)	-0.105 (-1.693, 1.482)	-0.117 (-2.124, 1.890)	-0.355 (-1.806, 1.095)	0.632 (-0.690, 1.955)	0.030 (-1.801, 1.860)	-0.304 (-2.152, 1.543)	-0.259 (-1.536, 1.017)
	cTBS (L-M1)	-0.012 (-1.826, 1.802)	-0.250 (-1.418, 0.918)	0.738 (-0.267, 1.742)	0.135 (-1.481, 1.750)	-0.199 (-1.834, 1.436)	-0.154 (-1.097, 0.790)
		cTBS (R-IPL)	-0.238 (-1.933, 1.457)	0.750 (-0.837, 2.336)	0.147 (-1.883, 2.176)	-0.187 (-2.232, 1.858)	-0.142 (-1.691, 1.407)
			iTBS (CV)	**0.988 (0.218, 1.757)**	0.385 (-1.096, 1.866)	0.051 (-1.451, 1.553)	0.096 (-0.592, 0.784)
				iTBS (L-DLPFC)	-0.603 (-1.959, 0.753)	-0.937 (-2.316, 0.443)	**-0.891 (-1.237, -0.546)**
					iTBS (L-SMA)	-0.334 (-2.206, 1.538)	-0.289 (-1.600, 1.023)
						iTBS (R-DLPFC)	0.045 (-1.290, 1.381)
							Sham

Comparisons between treatments should be read from left to right, and the estimate is in the cell in common between the column-defining treatment and the row-defining treatment. In the upper right half, SMDs <0 favor the row-defining treatment.

Global heterogeneity

As previously suggested (Huhn 2019), the common τ^2 was compared to the empirical distributions of heterogeneity found in the meta-analyses of pharmacological treatments for mental health outcomes, with a median of the τ^2 distribution of 0.049 and an inter-quartile range of 0.010 to 0.242 (Rhodes 2015), and the heterogeneity was considered low when the estimated τ^2 was below the 25% quartile, moderate when between 25% and 50% of the quartile, and high when above the 50% quartile.

Huhn M, et al. Lancet 2019;394(10202):939-51

Rhodes KM, et al. J Clin Epidemiol 2015;68(1):52-60

Between study variance (τ^2): 0.356 (heterogeneity assessment: high)

© 2024 Kishi T et al. *JAMA Network Open.*

Funnel plot (only sham-controlled trials)

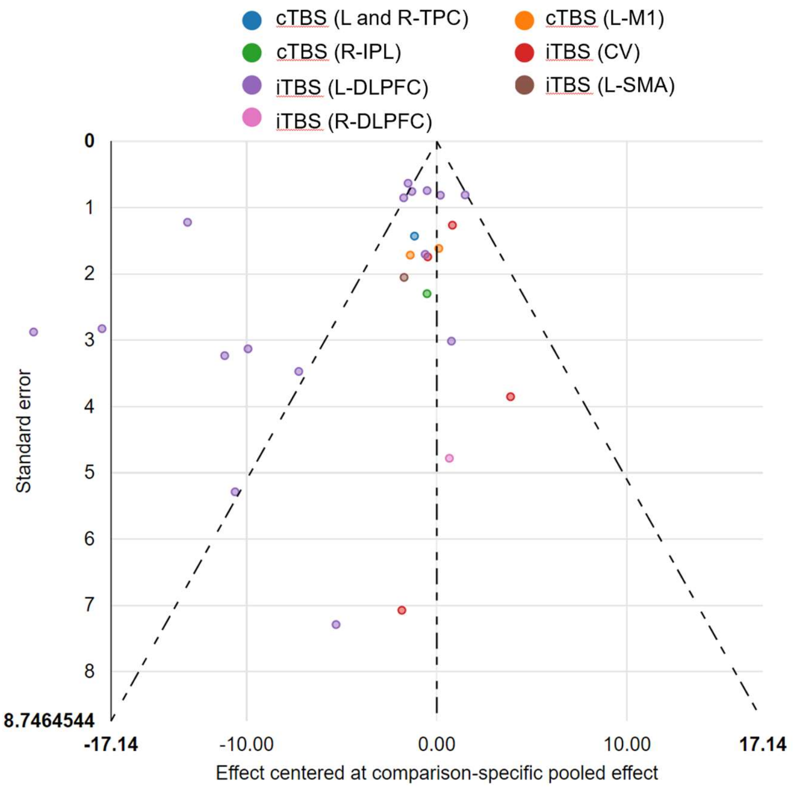

At least 10 studies included no comparisons other than iTBS (L-DLPFC); however, the funnel plots of the primary outcome revealed symmetry.

© 2024 Kishi T et al. *JAMA Network Open.*

Meta-regression analysis (the sham was the control)

Potential moderators	β (SE)
Individuals with predominantly negative symptoms (studies including individuals with predominantly negative symptoms vs. other studies) (k = 26)	-0.550 (0.410)
Proportion of females (k = 21)	-0.415 (26.334)
Mean age (k = 26)	0.432 (21.964)
Total number of participants (k = 26)	0.133 (23.242)
Antipsychotic dose (k = 16)	-0.502 (0.899)
Coil localization/targeting method (studies using magnetic resonance imaging [MRI] vs. studies not using MRI) (k = 19)	0.135 (22.291)
TBS coil (studies using figure 8 coils vs. studies using circulator coils) (k = 19)	-0.086 (11.043)
Use of sham coils (studies using sham coils vs. studies not using sham coils) (k = 20)	-0.070 (12.125)
Percent motor threshold (k = 26)	0.579 (0.435)
Number of sessions during a day (k = 25)	0.222 (16.867)
Number of sessions during a trial (k = 25)	-0.293 (15.463)
Number of pulses during a session (k = 23)	-0.373 (10.445)
Number of pulses during a trial (k = 23)	-0.654 (16.258)
Negative symptoms scales (studies using SANS vs. studies using PANSS) (k = 26)	-0.377 (10.748)
Publication year (k = 26)	0.703 (0.435)
Country where the trial was conducted (studies conducted in China vs. studies conducted in other countries) (k = 26)	0.153 (30.716)
Overall risk of bias (low risk or some concerns studies vs high risk studies) (k = 26)	0.477 (0.433)

The network meta-regression analyses revealed no potential confounding factors associated with the effect size of the primary outcome.

© 2024 Kishi T et al. *JAMA Network Open.*

Sensitivity analysis excluding studies which were evaluated overall risk of bias as high risk.

18 studies, 840 participants

League table (SMD with 95% confidence interval)

cTBS (L-M1)	-0.296 (-1.502, 0.909)	0.876 (-0.156, 1.909)	0.135 (-1.483, 1.753)	-0.199 (-1.837, 1.439)	-0.154 (-1.099, 0.791)
	iTBS (CV)	**1.173 (0.318, 2.028)**	0.431 (-1.080, 1.943)	0.097 (-1.435, 1.630)	0.143 (-0.605, 0.891)
		iTBS (L-DLPFC)	-0.742 (-2.119, 0.636)	-1.076 (-2.476, 0.325)	**-1.030 (-1.445, -0.616)**
			iTBS (L-SMA)	-0.334 (-2.209, 1.541)	-0.289 (-1.602, 1.025)
				iTBS (R-DLPFC)	0.045 (-1.292, 1.383)
					Sham

Between study variance (τ^2): 0.357 (heterogeneity assessment: high)

© 2024 Kishi T et al. *JAMA Network Open.*

Subgroup analysis involving only trials that included individuals with predominantly negative symptoms.

10 studies, 499 participants

League table (SMD with 95% confidence interval)

iTBS (CV)	1.533 (-0.820, 3.885)	0.551 (-2.554, 3.656)	0.217 (-2.898, 3.333)	0.263 (-1.922, 2.447)
	iTBS (L-DLPFC)	-0.982 (-3.354, 1.391)	-1.315 (-3.701, 1.070)	**-1.270 (-2.142, -0.398)**
		iTBS (L-SMA)	-0.334 (-3.464, 2.796)	-0.289 (-2.495, 1.918)
			iTBS (R-DLPFC)	0.045 (-2.175, 2.266)
				Sham

Between study variance (τ^2): 1.175 (heterogeneity assessment: high)

© 2024 Kishi T et al. *JAMA Network Open.*

CINeMA confidence rating

CINeMA is a web application that simplifies the evaluation of confidence in the findings from a network meta-analysis. CINeMA is based on a methodological framework described in the following articles, which consider the following six domains: within-study bias, reporting bias, indirectness, imprecision, heterogeneity, and incoherence. CINeMA grades the confidence in the results of each treatment comparison as high, moderate, low, or very low. If the comparison had only indirect evidence, the comparison was downgraded one level.

Nikolakopoulou A, et al., PLOS Medicine 2020 17 1-19, Papakonstantinou T, et al., Campbell Systematic Reviews 2020 16 e1080

(1) Within-study bias: Risk of bias in RCTs for the main outcomes was assessed independently using the Cochrane risk-of-bias tool for randomized trials (RoB 2).

(2) Reporting bias: Comparison-adjusted funnel plots with less than 10 studies are not meaningful. Therefore, all comparisons other than iTBS (L-DLPFC) vs sham were "Suspected."

(3) Indirectness: No indirectness was assumed. Selected rule: Average

(4) Imprecision: For sham comparisons the clinically meaningful threshold was set at a odds ratio of higher or lower than 1. For sham comparisons the clinically meaningful threshold was set at a standardized mean difference of higher or lower than 0. For comparisons of two TBS treatments the clinically meaningful threshold was set at standardized mean differences of -0.1 and 0.1 for continuous outcomes and at a odds ratio of 0.8 and 1.25
for dichotomous outcomes.

(5) Heterogeneity: We used recommendations automatically provided by CINeMA.

(6) Incoherence: We used recommendations automatically provided by CINeMA.

Comparison	Number of studies	Within-study bias	Reporting bias	Indirectness	Imprecision	Heterogeneity	Incoherence	Confidence rating
cTBS (L and R-TPC) vs Sham	1	Major concerns	Some concerns	No concerns	Major concerns	No concerns	Major concerns	Very low
cTBS (L-M1) vs Sham	2	Some concerns	Some concerns	No concerns	Major concerns	No concerns	Major concerns	Very low
cTBS (R-IPL) vs Sham	1	Major concerns	Some concerns	No concerns	Major concerns	No concerns	Major concerns	Very low
iTBS (CV) vs Sham	4	Some concerns	Some concerns	No concerns	Major concerns	No concerns	Major concerns	Very low
iTBS (L-DLPFC) vs Sham	16	Some concerns	No concerns	No concerns	No concerns	Major concerns	Major concerns	Low
iTBS (L-SMA) vs Sham	1	No concerns	Some concerns	No concerns	Major concerns	No concerns	Major concerns	Very low
iTBS (R-DLPFC) vs Sham	1	Some concerns	Some concerns	No concerns	Major concerns	No concerns	Major concerns	Very low
cTBS (L and R-TPC) vs cTBS (L-M1)	0	Major concerns	Some concerns	No concerns	Major concerns	No concerns	Major concerns	Very low

© 2024 Kishi T et al. *JAMA Network Open.*

cTBS (L and R-TPC) vs cTBS (R-IPL)	0	Major concerns	Some concerns	No concerns	Major concerns	No concerns	Major concerns	Very low
cTBS (L and R-TPC) vs iTBS (CV)	0	Some concerns	Some concerns	No concerns	Major concerns	No concerns	Major concerns	Very low
cTBS (L and R-TPC) vs iTBS (L-DLPFC)	0	Major concerns	Some concerns	No concerns	Major concerns	No concerns	Major concerns	Very low
cTBS (L and R-TPC) vs iTBS (L-SMA)	0	Some concerns	Some concerns	No concerns	Major concerns	No concerns	Major concerns	Very low
cTBS (L and R-TPC) vs iTBS (R-DLPFC)	0	Major concerns	Some concerns	No concerns	Major concerns	No concerns	Major concerns	Very low
cTBS (L-M1) vs cTBS (R-IPL)	0	Major concerns	Some concerns	No concerns	Major concerns	No concerns	Major concerns	Very low
cTBS (L-M1) vs iTBS (CV)	0	Some concerns	Some concerns	No concerns	Major concerns	No concerns	Major concerns	Very low
cTBS (L-M1) vs iTBS (L-DLPFC)	0	Some concerns	Some concerns	No concerns	Major concerns	No concerns	Major concerns	Very low
cTBS (L-M1) vs iTBS (L-SMA)	0	Some concerns	Some concerns	No concerns	Major concerns	No concerns	Major concerns	Very low
cTBS (L-M1) vs iTBS (R-DLPFC)	0	Some concerns	Some concerns	No concerns	Major concerns	No concerns	Major concerns	Very low
cTBS (R-IPL) vs iTBS (CV)	0	Some concerns	Some concerns	No concerns	Major concerns	No concerns	Major concerns	Very low
cTBS (R-IPL) vs iTBS (L-DLPFC)	0	Major concerns	Some concerns	No concerns	Major concerns	No concerns	Major concerns	Very low
cTBS (R-IPL) vs iTBS (L-SMA)	0	Some concerns	Some concerns	No concerns	Major concerns	No concerns	Major concerns	Very low
cTBS (R-IPL) vs iTBS (R-DLPFC)	0	Major concerns	Some concerns	No concerns	Major concerns	No concerns	Major concerns	Very low
iTBS (CV) vs iTBS (L-DLPFC)	0	Some concerns	Some concerns	No concerns	No concerns	Major concerns	Major concerns	Very low
iTBS (CV) vs iTBS (L-SMA)	0	No concerns	Some concerns	No concerns	Major concerns	No concerns	Major concerns	Very low
iTBS (CV) vs iTBS (R-DLPFC)	0	Some concerns	Some concerns	No concerns	Major concerns	No concerns	Major concerns	Very low
iTBS (L-DLPFC) vs iTBS (L-SMA)	0	Some concerns	Some concerns	No concerns	Major concerns	No concerns	Major concerns	Very low
iTBS (L-DLPFC) vs iTBS (R-DLPFC)	0	Some concerns	Some concerns	No concerns	Major concerns	No concerns	Major concerns	Very low
iTBS (L-SMA) vs iTBS (R-DLPFC)	0	Some concerns	Some concerns	No concerns	Major concerns	No concerns	Major concerns	Very low

© 2024 Kishi T et al. *JAMA Network Open.*

Pairwise meta-analysis for iTBS (L-DLPFC) vs. sham for negative symptoms.

16 studies, 719 participants

Forest plot

Study name	Std diff in means	Standard error	Variance	Lower limit	Upper limit	Z-Value	p-Value
Bation 2021	-0.032	0.428	0.183	-0.871	0.808	-0.074	0.941
Chen 2011	-1.001	0.329	0.108	-1.646	-0.357	-3.046	0.002
Gan 2014	-0.308	0.314	0.099	-0.924	0.308	-0.979	0.328
Jin 2021	0.138	0.224	0.050	-0.300	0.577	0.618	0.537
Jin 2023	-0.507	0.256	0.066	-1.009	-0.005	-1.980	0.048
Kazemi 2012	-4.164	1.126	1.267	-6.370	-1.958	-3.699	0.000
Mao 2019	-1.064	0.291	0.085	-1.634	-0.494	-3.657	0.000
Sun 2017	-2.310	0.258	0.067	-2.816	-1.804	-8.946	0.000
Vergallito 2024	-0.527	0.700	0.490	-1.899	0.845	-0.753	0.451
Wang 2020	-0.987	0.300	0.090	-1.574	-0.400	-3.295	0.001
Wang 2022	-0.986	0.277	0.077	-1.530	-0.442	-3.553	0.000
Wu 2021	-0.840	0.369	0.136	-1.563	-0.118	-2.279	0.023
Zhang 2010	-0.856	0.404	0.164	-1.648	-0.063	-2.116	0.034
Zhao 2014	-2.274	0.379	0.143	-3.015	-1.532	-6.006	0.000
Zhao 2021	-0.879	0.302	0.091	-1.471	-0.286	-2.907	0.004
Zheng 2012	-0.319	0.340	0.116	-0.986	0.348	-0.938	0.348
	-0.935	0.202	0.041	-1.330	-0.540	-4.638	0.000

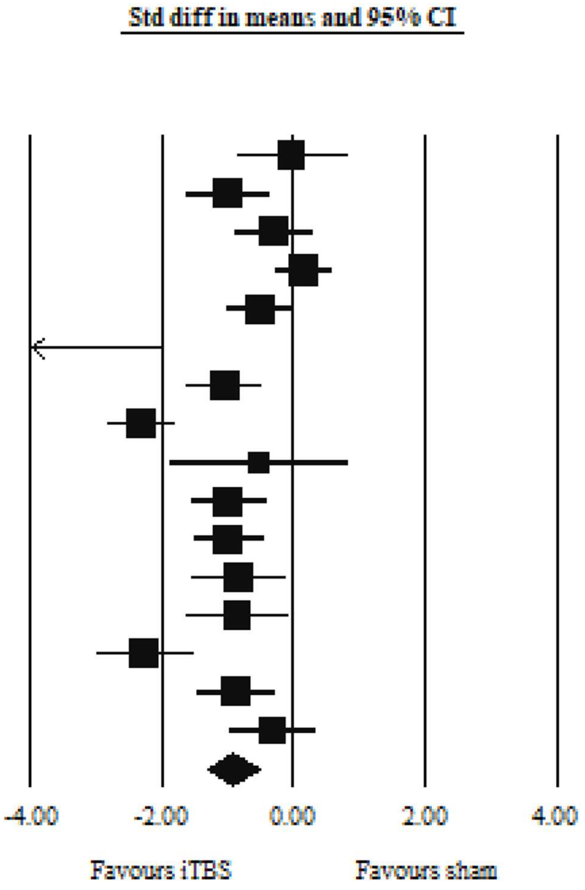

$I^2 = 82.67\%$

© 2024 Kishi T et al. *JAMA Network Open.*

Funnel plot

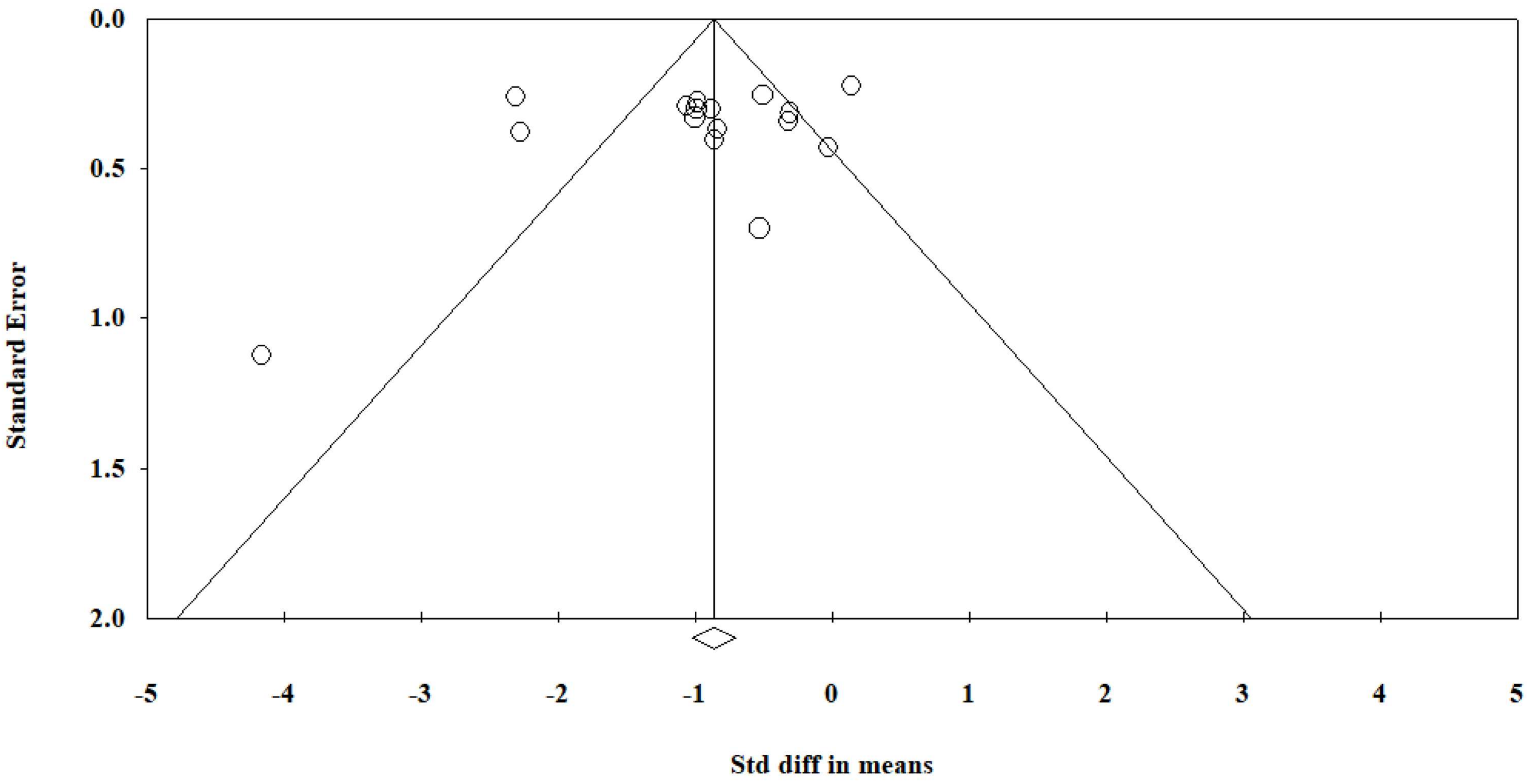

The funnel plots of the primary outcome revealed symmetry.

Egger's test

P = 0.355

© 2024 Kishi T et al. *JAMA Network Open.*

The univariate meta-regression analysis: iTBS (L-DLPFC) for negative symptoms.

	Coefficient	95% CI	p	I^2 (%)
Individuals with predominantly negative symptoms (studies including individuals with predominantly negative symptoms vs. other studies) (k = 16)	-0.565	-1.376, 0.246	0.172	82.892
Proportion of females (k = 11)	-0.010	-0.032, 0.012	0.386	76.534
Mean age (k = 16)	0.002	-0.031, 0.034	0.926	83.795
Total number of participants (k = 16)	-0.005	-0.023, 0.014	0.628	82.831
Antipsychotic dose (k = 8)*	-0.002	-0.004, -0.000	**0.045**	40.573
Coil localization/targeting method (studies using magnetic resonance imaging [MRI] vs. studies not using MRI) (k = 9)	-0.132	-0.884, 0.620	0.731	57.080
TBS coil (studies using figure 8 coils vs. studies using circulator coils) (k = 11)	-0.050	-1.039, 0.939	0.921	80.823
Use of sham coils (studies using sham coils vs. studies not using sham coils) (k = 15)	0.280	-0.637, 1.197	0.549	84.542
Percent motor threshold (k = 16)	0.026	-0.003, 0.056	0.080	79.039
Number of sessions during a day (k = 15)	0.188	-0.291, 0.668	0.442	84.096
Number of sessions during a trial (k = 16)	-0.023	-0.056, 0.011	0.185	79.687
Number of pulses during a session (k = 13)	-0.000	-0.001, 0.000	0.064	70.980
Number of pulses during a trial (k = 13)*	-0.000	-0.000, -0.000	**0.004**	60.426
Intersession interval (k = 5)**	0.007	-0.001, 0.016	0.087	0.000
Negative symptoms scales (studies using SANS vs. studies using PANSS) (k = 16)*	-0.781	-1.448, -0.114	**0.022**	74.139
Publication year (k = 16)	0.004	-0.091, 0.100	0.928	83.819
country where the trial was conducted (studies conducted in China vs. studies conducted in other countries) (k = 16)	-0.046	-1.262, 1.171	0.941	83.658

© 2024 Kishi T et al. *JAMA Network Open.*

Overall risk of bias (low risk or some concerns vs high risk) (k = 16)	-0.480	-1.314, 0.354	0.260	81.311

95% CI: 95% confidence interval, N: number of studies

*The bubble plots are on the next page.

**The meta-regression analysis included only accelerated iTBS studies

Bubble plot: An association between antipsychotic dose and the effect size.

Studies that included individuals who received a higher antipsychotic dose were associated with a larger effect size for the improvement of negative symptom scores compared to studies that included individuals who received a lower antipsychotic dose.

© 2024 Kishi T et al. *JAMA Network Open.*

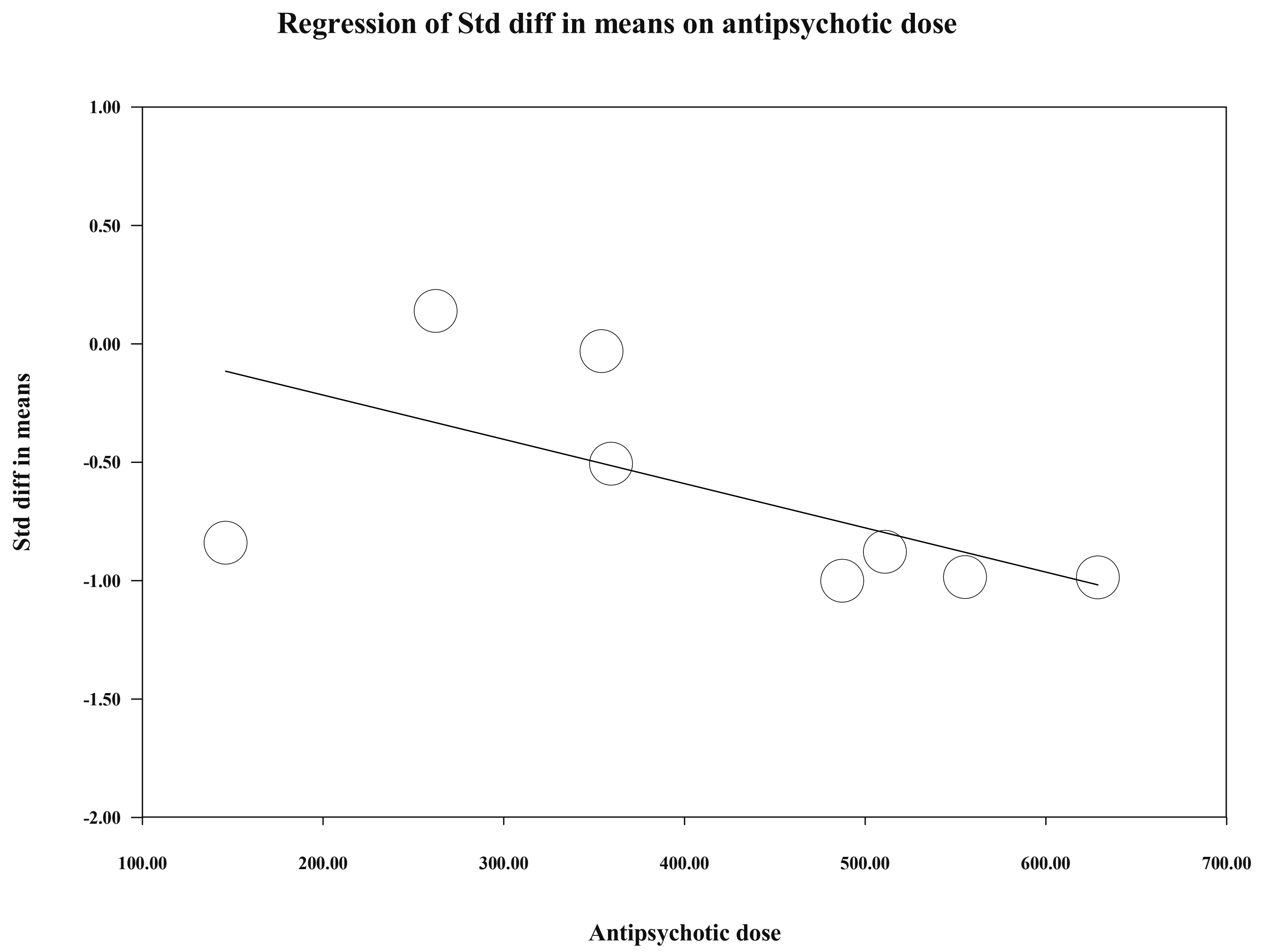

Bubble plot: An association between number of pulses during a trial and the effect size.
Studies with more pulses during a trial were associated with a larger effect size for the improvement of negative symptoms scores compared to studies with fewer pulses during a trial.

© 2024 Kishi T et al. *JAMA Network Open.*

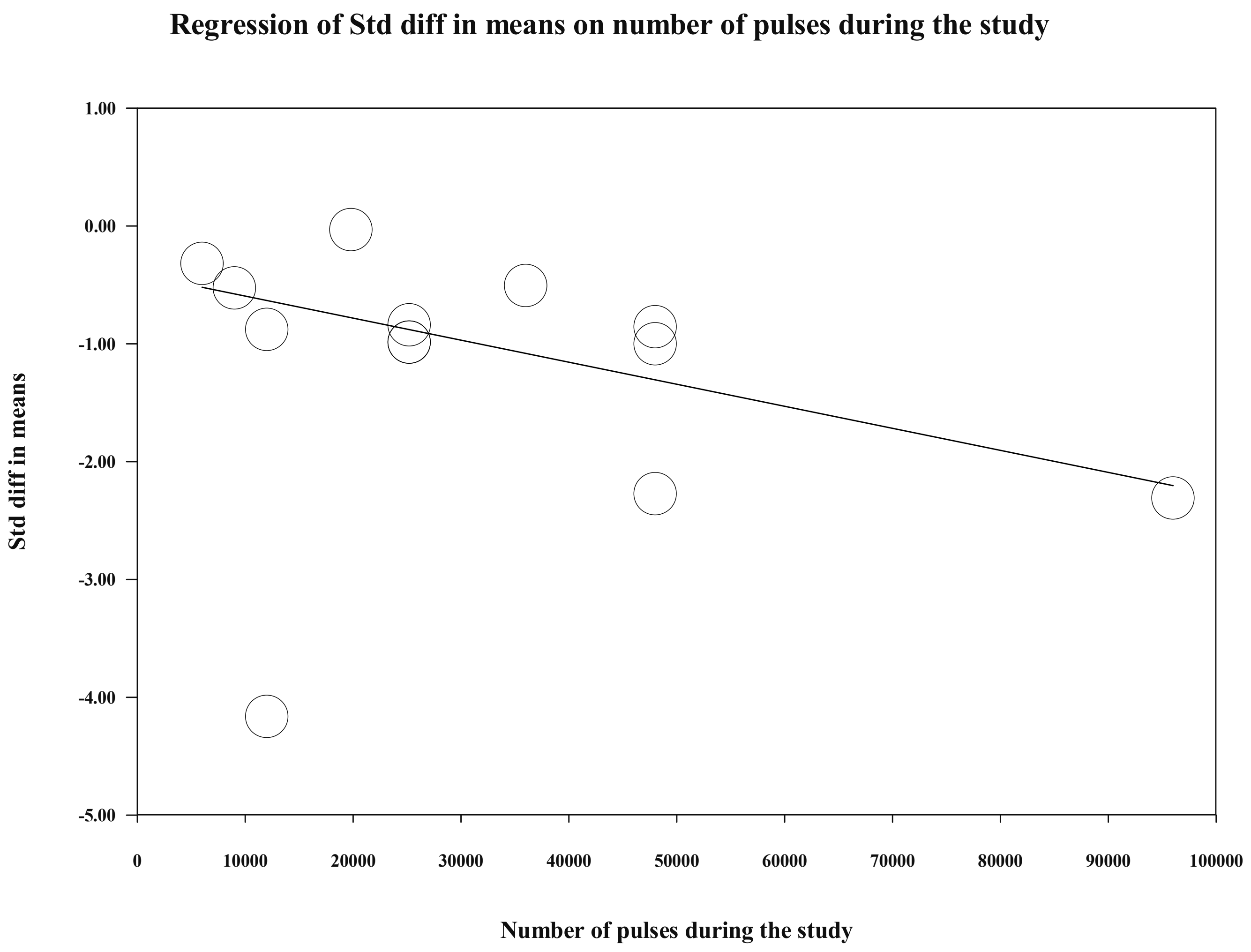

Bubble plot: An association between the difference in the scales (studies used PANSS-N or studies used SANS) and the effect size.
Studies that used SANS were associated with larger effect size for the improvement of negative symptom scores compared to studies that used PANSS.

© 2024 Kishi T et al. *JAMA Network Open.*

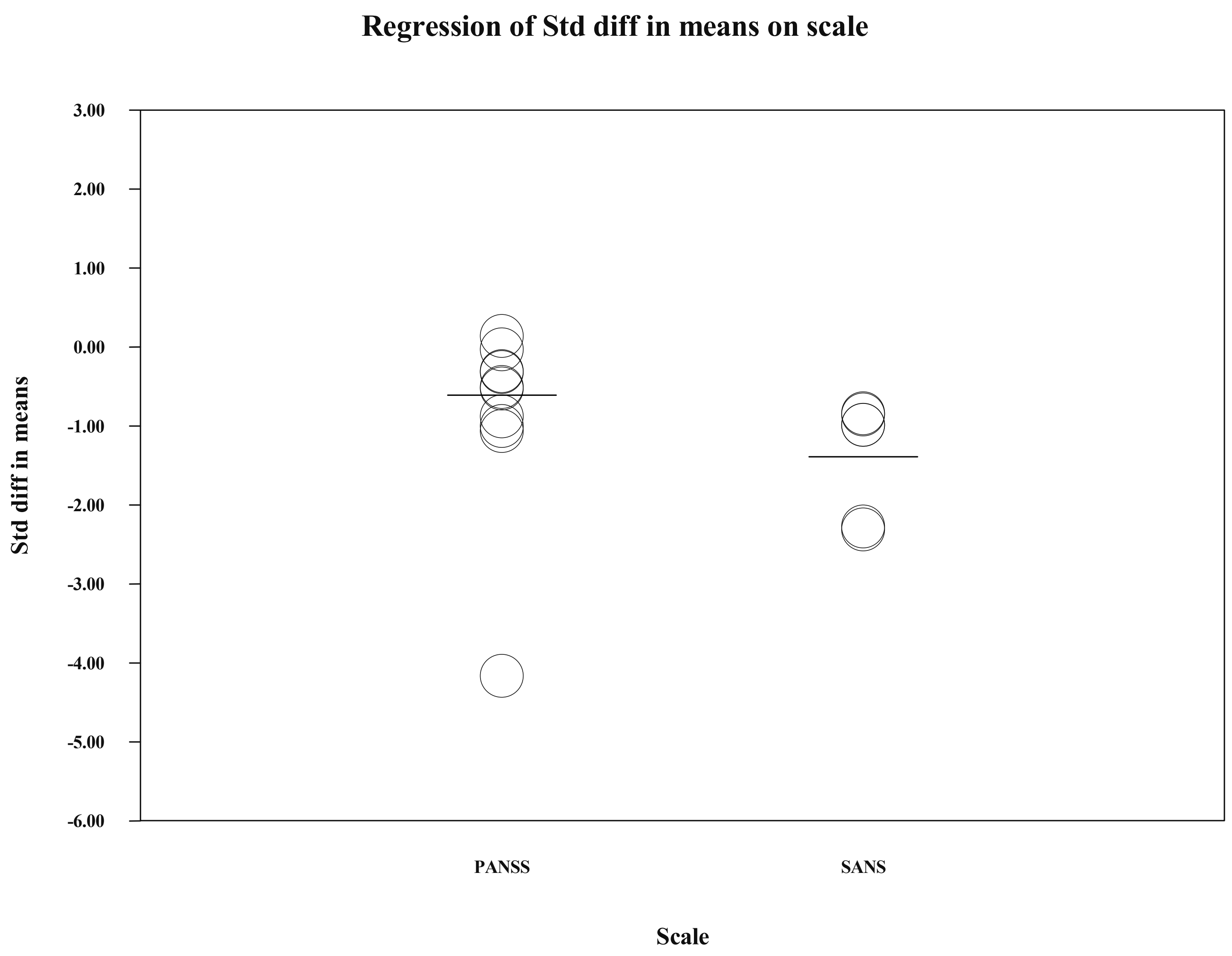

© 2024 Kishi T et al. *JAMA Network Open.*

eAppendix 2. Overall Symptoms

Network meta-analysis

20 studies, 826 participants

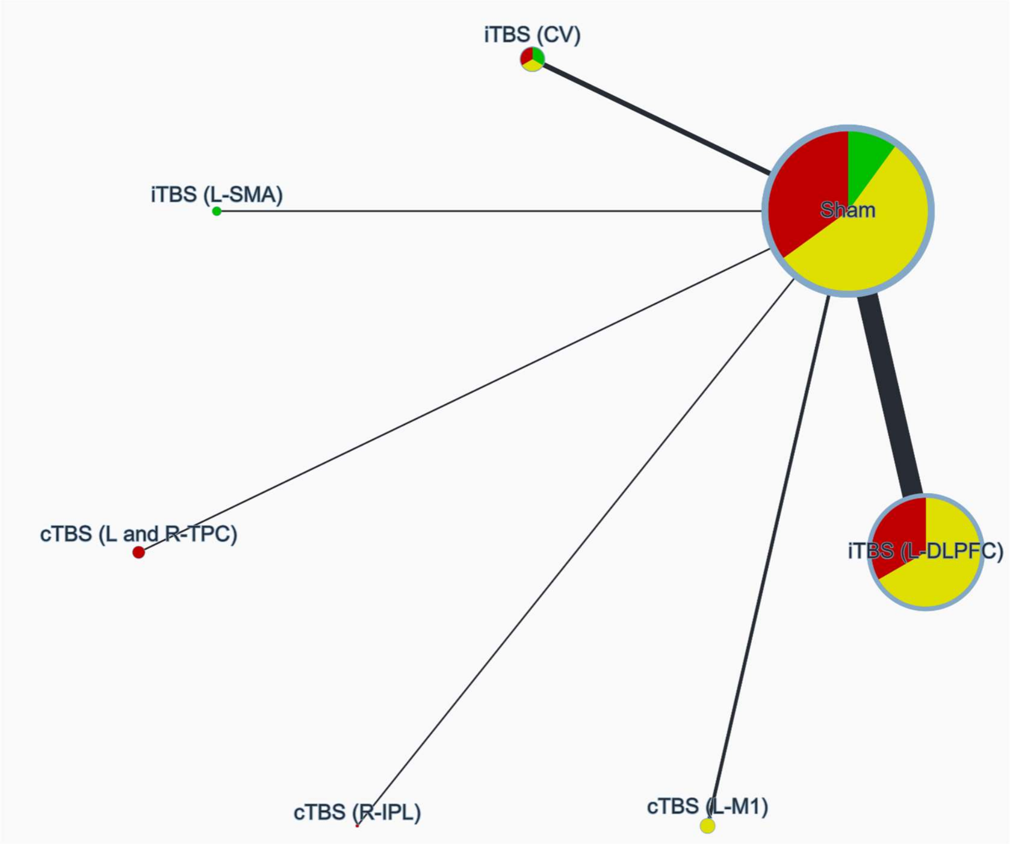

© 2024 Kishi T et al. *JAMA Network Open.*

League table (SMD with 95% confidence interval)

cTBS (L and R-TPC)	0.429 (-0.901, 1.759)	0.242 (-1.502, 1.987)	0.021 (-1.248, 1.289)	0.603 (-0.509, 1.714)	-0.176 (-1.702, 1.349)	-0.212 (-1.270, 0.846)
	cTBS (L-M1)	-0.186 (-1.790, 1.417)	-0.408 (-1.475, 0.659)	0.174 (-0.700, 1.048)	-0.605 (-1.967, 0.758)	-0.641 (-1.446, 0.165)
		cTBS (R-IPL)	-0.222 (-1.775, 1.331)	0.360 (-1.067, 1.788)	-0.418 (-2.188, 1.351)	-0.454 (-1.841, 0.932)
			iTBS (CV)	0.582 (-0.195, 1.359)	-0.197 (-1.499, 1.106)	-0.232 (-0.932, 0.467)
				iTBS (L-DLPFC)	-0.779 (-1.929, 0.371)	**-0.814 (-1.154, -0.475)**
					iTBS (L-SMA)	-0.036 (-1.135, 1.063)
						Sham

Comparisons between treatments should be read from left to right, and the estimate is in the cell in common between the column-defining treatment and the row-defining treatment. In the upper right half, SMDs <0 favor the row-defining treatment.

Global heterogeneity

Between study variance (τ^2): 0.223 (heterogeneity assessment: moderate to high)

© 2024 Kishi T et al. *JAMA Network Open.*

CINeMA confidence rating

Comparison	Number of studies	Within-study bias	Reporting bias	Indirectness	Imprecision	Heterogeneity	Incoherence	Confidence rating
cTBS (L and R-TPC) vs Sham	1	Major concerns	Some concerns	No concerns	Major concerns	No concerns	Major concerns	Very low
cTBS (L-M1) vs Sham	2	Some concerns	Some concerns	No concerns	Major concerns	No concerns	Major concerns	Very low
cTBS (R-IPL) vs Sham	1	Major concerns	Some concerns	No concerns	Major concerns	No concerns	Major concerns	Very low
iTBS (CV) vs Sham	3	Some concerns	Some concerns	No concerns	Major concerns	No concerns	Major concerns	Very low
iTBS (L-DLPFC) vs Sham	12	Some concerns	No concerns	No concerns	No concerns	Major concerns	Major concerns	Low
iTBS (L-SMA) vs Sham	1	No concerns	Some concerns	No concerns	Major concerns	No concerns	Major concerns	Very low
cTBS (L and R-TPC) vs cTBS (L-M1)	0	Major concerns	Some concerns	No concerns	Major concerns	No concerns	Major concerns	Very low
cTBS (L and R-TPC) vs cTBS (R-IPL)	0	Major concerns	Some concerns	No concerns	Major concerns	No concerns	Major concerns	Very low
cTBS (L and R-TPC) vs iTBS (CV)	0	Some concerns	Some concerns	No concerns	Major concerns	No concerns	Major concerns	Very low
cTBS (L and R-TPC) vs iTBS (L-DLPFC)	0	Major concerns	Some concerns	No concerns	Major concerns	No concerns	Major concerns	Very low
cTBS (L and R-TPC) vs iTBS (L-SMA)	0	Some concerns	Some concerns	No concerns	Major concerns	No concerns	Major concerns	Very low
cTBS (L-M1) vs cTBS (R-IPL)	0	Major concerns	Some concerns	No concerns	Major concerns	No concerns	Major concerns	Very low
cTBS (L-M1) vs iTBS (CV)	0	Some concerns	Some concerns	No concerns	Major concerns	No concerns	Major concerns	Very low
cTBS (L-M1) vs iTBS (L-DLPFC)	0	Some concerns	Some concerns	No concerns	Major concerns	No concerns	Major concerns	Very low
cTBS (L-M1) vs iTBS (L-SMA)	0	Some concerns	Some concerns	No concerns	Major concerns	No concerns	Major concerns	Very low
cTBS (R-IPL) vs iTBS (CV)	0	Some concerns	Some concerns	No concerns	Major concerns	No concerns	Major concerns	Very low
cTBS (R-IPL) vs iTBS (L-DLPFC)	0	Major concerns	Some concerns	No concerns	Major concerns	No concerns	Major concerns	Very low
cTBS (R-IPL) vs iTBS (L-SMA)	0	Some concerns	Some concerns	No concerns	Major concerns	No concerns	Major concerns	Very low
iTBS (CV) vs iTBS (L-DLPFC)	0	Some concerns	Some concerns	No concerns	Major concerns	No concerns	Major concerns	Very low
iTBS (CV) vs iTBS (L-SMA)	0	No concerns	Some concerns	No concerns	Major concerns	No concerns	Major concerns	Very low
iTBS (L-DLPFC) vs iTBS (L-SMA)	0	Some concerns	Some concerns	No concerns	Major concerns	No concerns	Major concerns	Very low

© 2024 Kishi T et al. *JAMA Network Open.*

eAppendix 3. Positive Symptoms

Network meta-analysis

24 studies, 1006 participants

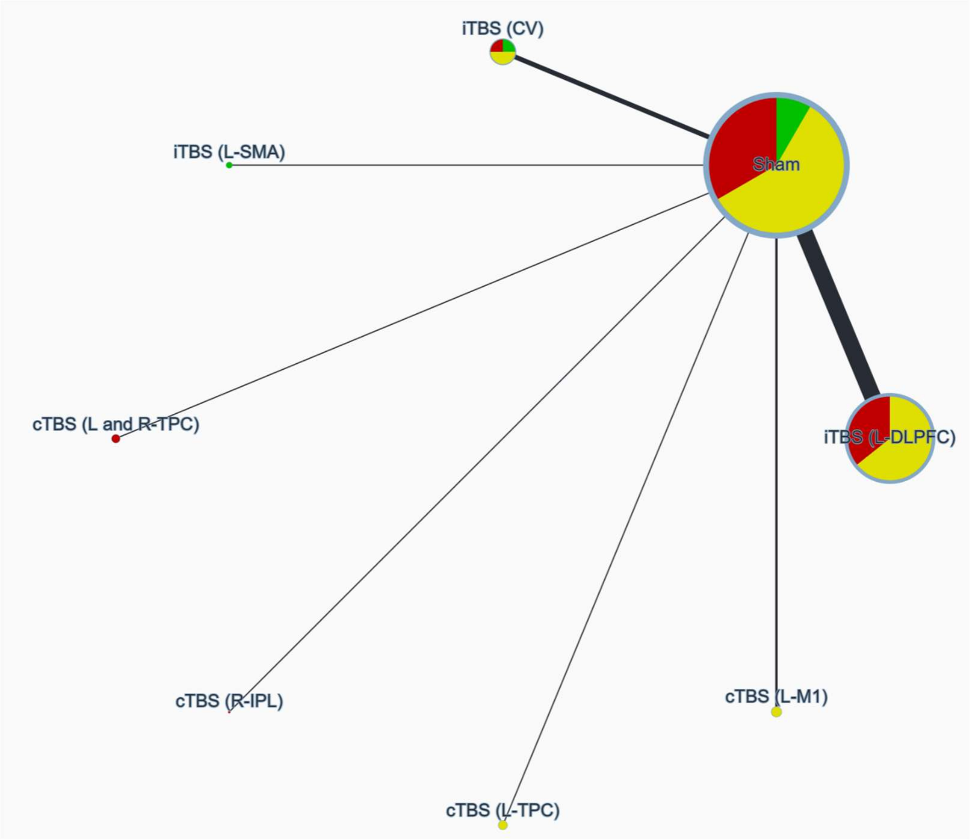

© 2024 Kishi T et al. *JAMA Network Open.*

League table (SMD with 95% confidence interval)

cTBS (L and R-TPC)	1.050 (-0.882, 2.982)	0.218 (-1.979, 2.416)	0.192 (-2.178, 2.562)	0.360 (-1.404, 2.123)	0.035 (-1.585, 1.655)	0.019 (-2.203, 2.241)	0.218 (-1.339, 1.776)
	cTBS (L-M1)	-0.832 (-2.758, 1.095)	-0.858 (-2.979, 1.263)	-0.690 (-2.102, 0.722)	-1.015 (-2.243, 0.213)	-1.031 (-2.986, 0.924)	-0.832 (-1.975, 0.312)
		cTBS (L-TPC)	-0.026 (-2.392, 2.339)	0.142 (-1.616, 1.899)	-0.183 (-1.797, 1.430)	-0.199 (-2.417, 2.018)	0.000 (-1.550, 1.550)
			cTBS (R-IPL)	0.168 (-1.801, 2.137)	-0.157 (-1.999, 1.685)	-0.173 (-2.561, 2.216)	0.026 (-1.760, 1.813)
				iTBS (CV)	-0.325 (-1.266, 0.616)	-0.341 (-2.129, 1.448)	-0.142 (-0.970, 0.686)
					iTBS (L-DLPFC)	-0.016 (-1.663, 1.632)	0.183 (-0.264, 0.631)
						iTBS (L-SMA)	0.199 (-1.386, 1.785)
							Sham

Comparisons between treatments should be read from left to right, and the estimate is in the cell in common between the column-defining treatment and the row-defining treatment. In the upper right half, SMDs <0 favor the row-defining treatment.

Global heterogeneity

Between study variance (τ^2): 0.563 (heterogeneity assessment: high)

© 2024 Kishi T et al. *JAMA Network Open.*

CINeMA confidence rating

Comparison	Number of studies	Within-study bias	Reporting bias	Indirectness	Imprecision	Heterogeneity	Incoherence	Confidence rating
cTBS (L and R-TPC) vs Sham	1	Major concerns	Some concerns	No concerns	Major concerns	No concerns	Major concerns	Very low
cTBS (L-M1) vs Sham	2	Some concerns	Some concerns	No concerns	Major concerns	No concerns	Major concerns	Very low
cTBS (L-TPC) vs Sham	1	Some concerns	Some concerns	No concerns	Major concerns	No concerns	Major concerns	Very low
cTBS (R-IPL) vs Sham	1	Major concerns	Some concerns	No concerns	Major concerns	No concerns	Major concerns	Very low
iTBS (CV) vs Sham	4	Some concerns	Some concerns	No concerns	Major concerns	No concerns	Major concerns	Very low
iTBS (L-DLPFC) vs Sham	14	Some concerns	No concerns	No concerns	Major concerns	No concerns	Major concerns	Low
iTBS (L-SMA) vs Sham	1	No concerns	Some concerns	No concerns	Major concerns	No concerns	Major concerns	Very low
cTBS (L and R-TPC) vs cTBS (L-M1)	0	Major concerns	Some concerns	No concerns	Major concerns	No concerns	Major concerns	Very low
cTBS (L and R-TPC) vs cTBS (L-TPC)	0	Major concerns	Some concerns	No concerns	Major concerns	No concerns	Major concerns	Very low
cTBS (L and R-TPC) vs cTBS (R-IPL)	0	Major concerns	Some concerns	No concerns	Major concerns	No concerns	Major concerns	Very low
cTBS (L and R-TPC) vs iTBS (CV)	0	Some concerns	Some concerns	No concerns	Major concerns	No concerns	Major concerns	Very low
cTBS (L and R-TPC) vs iTBS (L-DLPFC)	0	Major concerns	Some concerns	No concerns	Major concerns	No concerns	Major concerns	Very low
cTBS (L and R-TPC) vs iTBS (L-SMA)	0	Some concerns	Some concerns	No concerns	Major concerns	No concerns	Major concerns	Very low
cTBS (L-M1) vs cTBS (L-TPC)	0	Some concerns	Some concerns	No concerns	Major concerns	No concerns	Major concerns	Very low
cTBS (L-M1) vs cTBS (R-IPL)	0	Major concerns	Some concerns	No concerns	Major concerns	No concerns	Major concerns	Very low
cTBS (L-M1) vs iTBS (CV)	0	Some concerns	Some concerns	No concerns	Major concerns	No concerns	Major concerns	Very low
cTBS (L-M1) vs iTBS (L-DLPFC)	0	Some concerns	Some concerns	No concerns	Major concerns	No concerns	Major concerns	Very low
cTBS (L-M1) vs iTBS (L-SMA)	0	Some concerns	Some concerns	No concerns	Major concerns	No concerns	Major concerns	Very low
cTBS (L-TPC) vs cTBS (R-IPL)	0	Major concerns	Some concerns	No concerns	Major concerns	No concerns	Major concerns	Very low
cTBS (L-TPC) vs iTBS (CV)	0	Some concerns	Some concerns	No concerns	Major concerns	No concerns	Major concerns	Very low
cTBS (L-TPC) vs iTBS (L-DLPFC)	0	Some concerns	Some concerns	No concerns	Major concerns	No concerns	Major concerns	Very low
cTBS (L-TPC) vs iTBS (L-SMA)	0	Some concerns	Some concerns	No concerns	Major concerns	No concerns	Major concerns	Very low
cTBS (R-IPL) vs iTBS (CV)	0	Some concerns	Some concerns	No concerns	Major concerns	No concerns	Major concerns	Very low
cTBS (R-IPL) vs iTBS (L-DLPFC)	0	Major concerns	Some concerns	No concerns	Major concerns	No concerns	Major concerns	Very low
cTBS (R-IPL) vs iTBS (L-SMA)	0	Some concerns	Some concerns	No concerns	Major concerns	No concerns	Major concerns	Very low
iTBS (CV) vs iTBS (L-DLPFC)	0	Some concerns	Some concerns	No concerns	Major concerns	No concerns	Major concerns	Very low

© 2024 Kishi T et al. *JAMA Network Open.*

iTBS (CV) vs iTBS (L-SMA)	0	No concerns	Some concerns	No concerns	Major concerns	No concerns	Major concerns	Very low
iTBS (L-DLPFC) vs iTBS (L-SMA)	0	Some concerns	Some concerns	No concerns	Major concerns	No concerns	Major concerns	Very low

© 2024 Kishi T et al. *JAMA Network Open.*

eAppendix 4. PANSS General Subscale Score

<u>Network meta-analysis</u>

18 studies, 690 participants

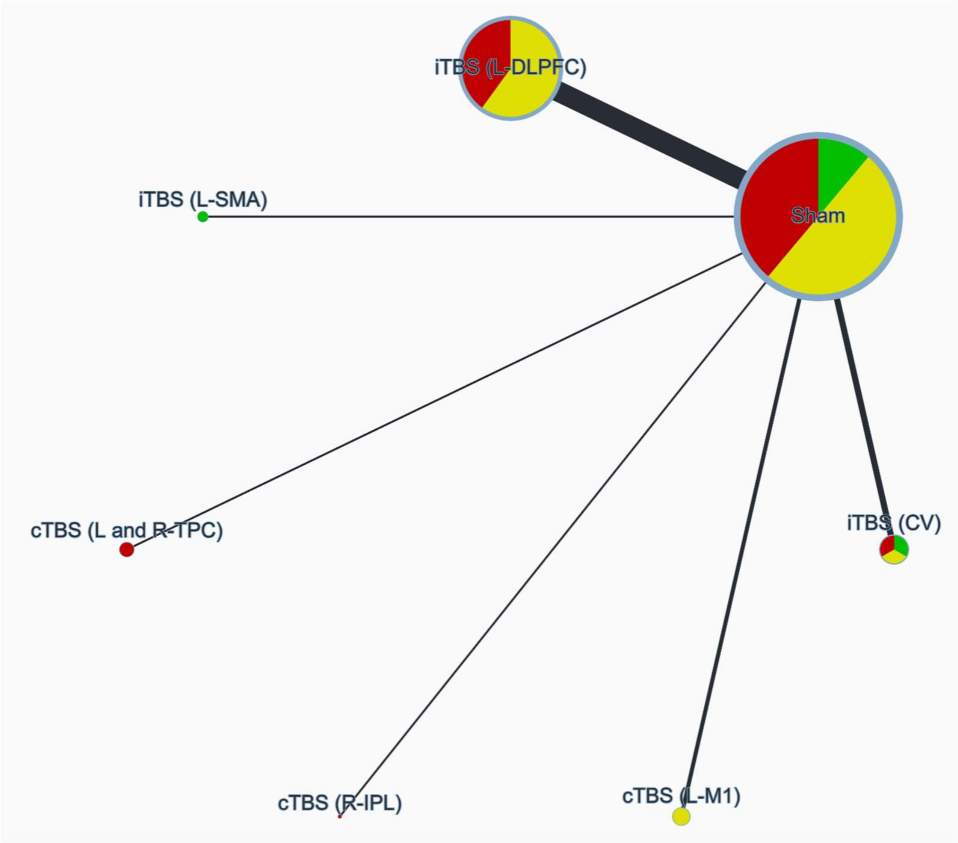

© 2024 Kishi T et al. *JAMA Network Open.*

League table (SMD with 95% confidence interval)

cTBS (L and R-TPC)	0.232 (-0.895, 1.359)	0.283 (-1.270, 1.837)	-0.107 (-1.181, 0.967)	0.242 (-0.702, 1.187)	-0.368 (-1.659, 0.924)	-0.329 (-1.219, 0.561)
	cTBS (L-M1)	0.051 (-1.398, 1.500)	-0.339 (-1.256, 0.577)	0.010 (-0.751, 0.772)	-0.600 (-1.764, 0.564)	-0.561 (-1.253, 0.131)
		cTBS (R-IPL)	-0.391 (-1.799, 1.018)	-0.041 (-1.353, 1.272)	-0.651 (-2.232, 0.929)	-0.612 (-1.886, 0.661)
			iTBS (CV)	0.350 (-0.331, 1.030)	-0.261 (-1.373, 0.852)	-0.222 (-0.823, 0.380)
				iTBS (L-DLPFC)	-0.610 (-1.599, 0.378)	**-0.571 (-0.889, -0.253)**
					iTBS (L-SMA)	0.039 (-0.897, 0.975)
						Sham

Comparisons between treatments should be read from left to right, and the estimate is in the cell in common between the column-defining treatment and the row-defining treatment. In the upper right half, SMDs <0 favor the row-defining treatment.

Global heterogeneity

Between study variance (τ^2): 0.137 (heterogeneity assessment: moderate to high)

© 2024 Kishi T et al. *JAMA Network Open.*

CINeMA confidence rating

Comparison	Number of studies	Within-study bias	Reporting bias	Indirectness	Imprecision	Heterogeneity	Incoherence	Confidence rating
cTBS (L and R-TPC) vs Sham	1	Major concerns	Some concerns	No concerns	Major concerns	No concerns	Major concerns	Very low
cTBS (L-M1) vs Sham	2	Some concerns	Some concerns	No concerns	Major concerns	No concerns	Major concerns	Very low
cTBS (R-IPL) vs Sham	1	Major concerns	Some concerns	No concerns	Major concerns	No concerns	Major concerns	Very low
iTBS (CV) vs Sham	3	Some concerns	Some concerns	No concerns	Major concerns	No concerns	Major concerns	Very low
iTBS (L-DLPFC) vs Sham	10	Some concerns	No concerns	No concerns	No concerns	Major concerns	Major concerns	Low
iTBS (L-SMA) vs Sham	1	No concerns	Some concerns	No concerns	Major concerns	No concerns	Major concerns	Very low
cTBS (L and R-TPC) vs cTBS (L-M1)	0	Major concerns	Some concerns	No concerns	Major concerns	No concerns	Major concerns	Very low
cTBS (L and R-TPC) vs cTBS (R-IPL)	0	Major concerns	Some concerns	No concerns	Major concerns	No concerns	Major concerns	Very low
cTBS (L and R-TPC) vs iTBS (CV)	0	Some concerns	Some concerns	No concerns	Major concerns	No concerns	Major concerns	Very low
cTBS (L and R-TPC) vs iTBS (L-DLPFC)	0	Major concerns	Some concerns	No concerns	Major concerns	No concerns	Major concerns	Very low
cTBS (L and R-TPC) vs iTBS (L-SMA)	0	Some concerns	Some concerns	No concerns	Major concerns	No concerns	Major concerns	Very low
cTBS (L-M1) vs cTBS (R-IPL)	0	Major concerns	Some concerns	No concerns	Major concerns	No concerns	Major concerns	Very low
cTBS (L-M1) vs iTBS (CV)	0	Some concerns	Some concerns	No concerns	Major concerns	No concerns	Major concerns	Very low
cTBS (L-M1) vs iTBS (L-DLPFC)	0	Some concerns	Some concerns	No concerns	Major concerns	No concerns	Major concerns	Very low
cTBS (L-M1) vs iTBS (L-SMA)	0	Some concerns	Some concerns	No concerns	Major concerns	No concerns	Major concerns	Very low
cTBS (R-IPL) vs iTBS (CV)	0	Some concerns	Some concerns	No concerns	Major concerns	No concerns	Major concerns	Very low
cTBS (R-IPL) vs iTBS (L-DLPFC)	0	Major concerns	Some concerns	No concerns	Major concerns	No concerns	Major concerns	Very low
cTBS (R-IPL) vs iTBS (L-SMA)	0	Some concerns	Some concerns	No concerns	Major concerns	No concerns	Major concerns	Very low
iTBS (CV) vs iTBS (L-DLPFC)	0	Some concerns	Some concerns	No concerns	Major concerns	No concerns	Major concerns	Very low
iTBS (CV) vs iTBS (L-SMA)	0	No concerns	Some concerns	No concerns	Major concerns	No concerns	Major concerns	Very low
iTBS (L-DLPFC) vs iTBS (L-SMA)	0	Some concerns	Some concerns	No concerns	Major concerns	No concerns	Major concerns	Very low

© 2024 Kishi T et al. *JAMA Network Open.*

eAppendix 5. Depressive Symptoms

Network meta-analysis

8 studies, 350 participants

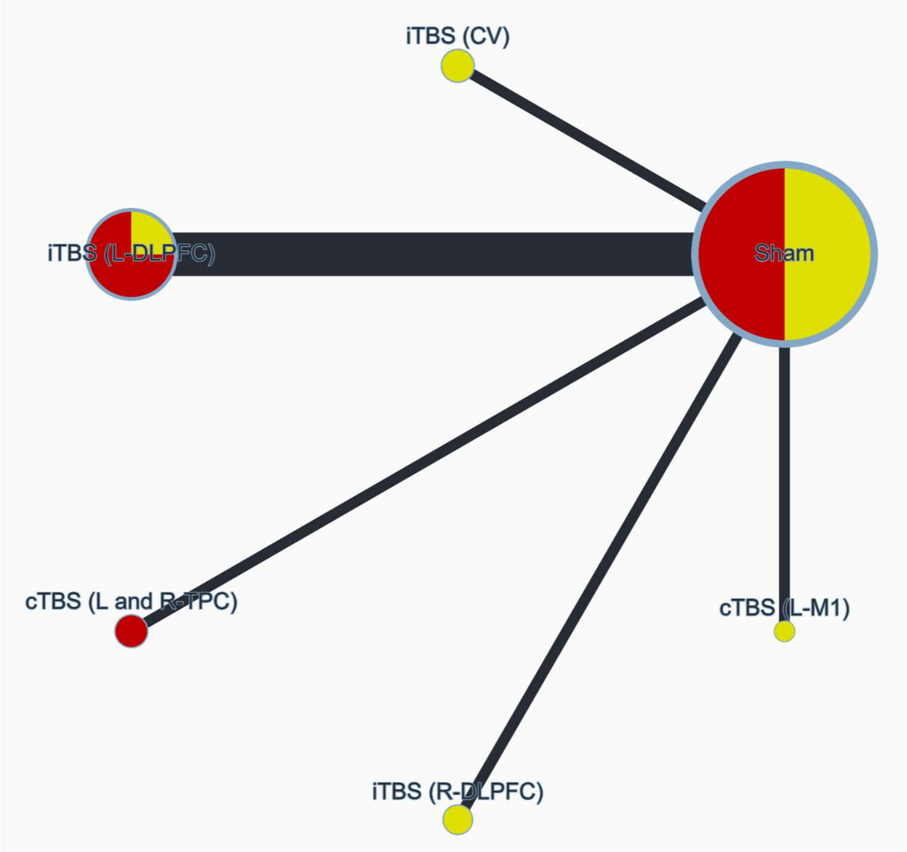

© 2024 Kishi T et al. *JAMA Network Open.*

League table (SMD with 95% confidence interval)

cTBS (L and R-TPC)	0.291 (-0.523, 1.104)	0.023 (-0.696, 0.742)	**0.629 (0.020, 1.239)**	-0.140 (-0.964, 0.683)	-0.075 (-0.586, 0.436)
	cTBS (L-M1)	-0.268 (-1.079, 0.543)	0.339 (-0.377, 1.054)	-0.431 (-1.336, 0.474)	-0.366 (-0.999, 0.268)
		iTBS (CV)	**0.606 (0.001, 1.212)**	-0.163 (-0.984, 0.657)	-0.098 (-0.604, 0.409)
			iTBS (L-DLPFC)	**-0.770 (-1.496, -0.044)**	**-0.704 (-1.037, -0.372)**
				iTBS (R-DLPFC)	0.066 (-0.580, 0.711)
					Sham

Comparisons between treatments should be read from left to right, and the estimate is in the cell in common between the column-defining treatment and the row-defining treatment. In the upper right half, SMDs <0 favor the row-defining treatment.

Global heterogeneity

Between study variance (τ^2): 0.000 (heterogeneity assessment: low)

© 2024 Kishi T et al. *JAMA Network Open.*

CINeMA confidence rating

Comparison	Number of studies	Within-study bias	Reporting bias	Indirectness	Imprecision	Heterogeneity	Incoherence	Confidence rating
cTBS (L and R-TPC) vs Sham	1	Major concerns	Some concerns	No concerns	Major concerns	No concerns	Major concerns	Very low
cTBS (L-M1) vs Sham	1	Some concerns	Some concerns	No concerns	Major concerns	No concerns	Major concerns	Very low
iTBS (CV) vs Sham	1	Some concerns	Some concerns	No concerns	Major concerns	No concerns	Major concerns	Very low
iTBS (L-DLPFC) vs Sham	4	Major concerns	Some concerns	No concerns	No concerns	Major concerns	Major concerns	Very low
iTBS (R-DLPFC) vs Sham	1	Some concerns	Some concerns	No concerns	Major concerns	No concerns	Major concerns	Very low
cTBS (L and R-TPC) vs cTBS (L-M1)	0	Major concerns	Some concerns	No concerns	Major concerns	No concerns	Major concerns	Very low
cTBS (L and R-TPC) vs iTBS (CV)	0	Major concerns	Some concerns	No concerns	Major concerns	No concerns	Major concerns	Very low
cTBS (L and R-TPC) vs iTBS (L-DLPFC)	0	Major concerns	Some concerns	No concerns	No concerns	Major concerns	Major concerns	Very low
cTBS (L and R-TPC) vs iTBS (R-DLPFC)	0	Major concerns	Some concerns	No concerns	Major concerns	No concerns	Major concerns	Very low
cTBS (L-M1) vs iTBS (CV)	0	Some concerns	Some concerns	No concerns	Major concerns	No concerns	Major concerns	Very low
cTBS (L-M1) vs iTBS (L-DLPFC)	0	Some concerns	Some concerns	No concerns	Major concerns	No concerns	Major concerns	Very low
cTBS (L-M1) vs iTBS (R-DLPFC)	0	Some concerns	Some concerns	No concerns	Major concerns	No concerns	Major concerns	Very low
iTBS (CV) vs iTBS (L-DLPFC)	0	Some concerns	Some concerns	No concerns	No concerns	Major concerns	Major concerns	Very low
iTBS (CV) vs iTBS (R-DLPFC)	0	Some concerns	Some concerns	No concerns	Major concerns	No concerns	Major concerns	Very low
iTBS (L-DLPFC) vs iTBS (R-DLPFC)	0	Some concerns	Some concerns	No concerns	No concerns	Major concerns	Major concerns	Very low

© 2024 Kishi T et al. *JAMA Network Open.*

eAppendix 6. Anxiety Symptoms

Network meta-analysis

4 studies, 180 participants

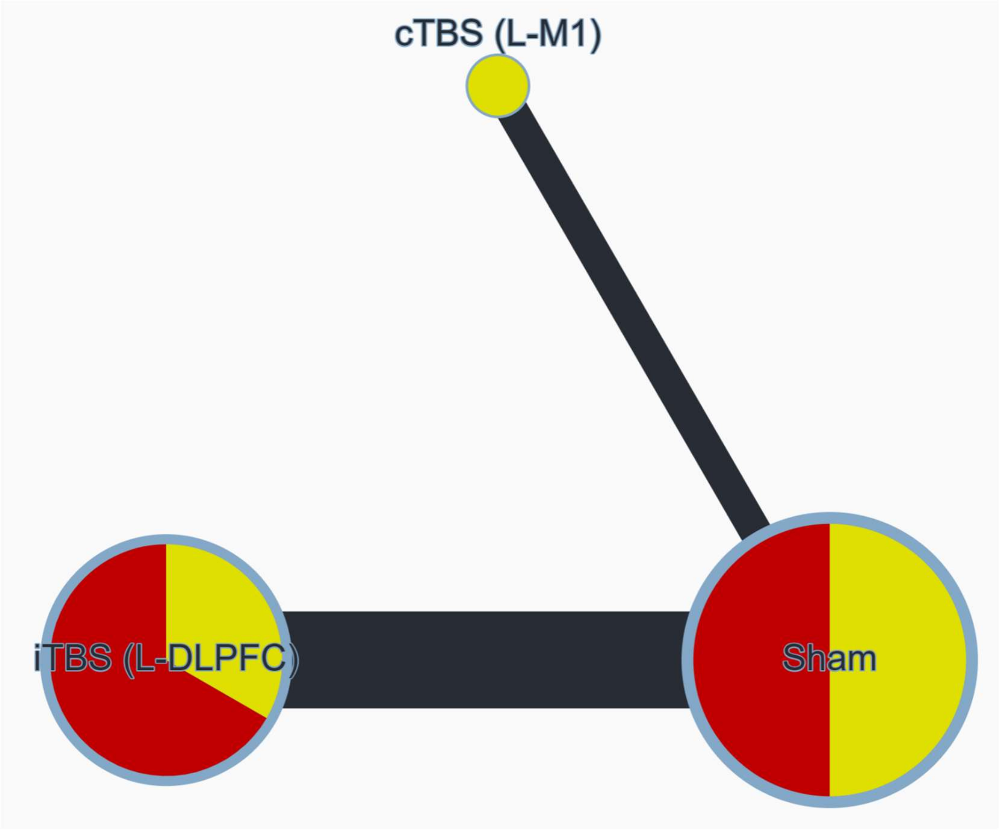

© 2024 Kishi T et al. *JAMA Network Open.*

League table (SMD with 95% confidence interval)

cTBS (L-M1)	0.596 (-0.118, 1.309)	0.012 (-0.616, 0.640)
	iTBS (L-DLPFC)	**-0.583 (-0.922, -0.244)**
		Sham

Comparisons between treatments should be read from left to right, and the estimate is in the cell in common between the column-defining treatment and the row-defining treatment. In the upper right half, SMDs <0 favor the row-defining treatment.

Global heterogeneity

Between study variance (τ^2): 0.000 (heterogeneity assessment: low)

© 2024 Kishi T et al. *JAMA Network Open.*

CINeMA confidence rating

Comparison	Number of studies	Within-study bias	Reporting bias	Indirectness	Imprecision	Heterogeneity	Incoherence	Confidence rating
cTBS (L-M1) vs Sham	1	Some concerns	Some concerns	No concerns	Major concerns	No concerns	Major concerns	Very low
iTBS (L-DLPFC) vs Sham	3	Major concerns	Some concerns	No concerns	No concerns	Major concerns	Major concerns	Very low
cTBS (L-M1) vs iTBS (L-DLPFC)	0	Some concerns	Some concerns	No concerns	Major concerns	No concerns	Major concerns	Very low

© 2024 Kishi T et al. *JAMA Network Open.*

eAppendix 7. Overall Cognitive Impairment

Network meta-analysis

5 studies, 214 participants

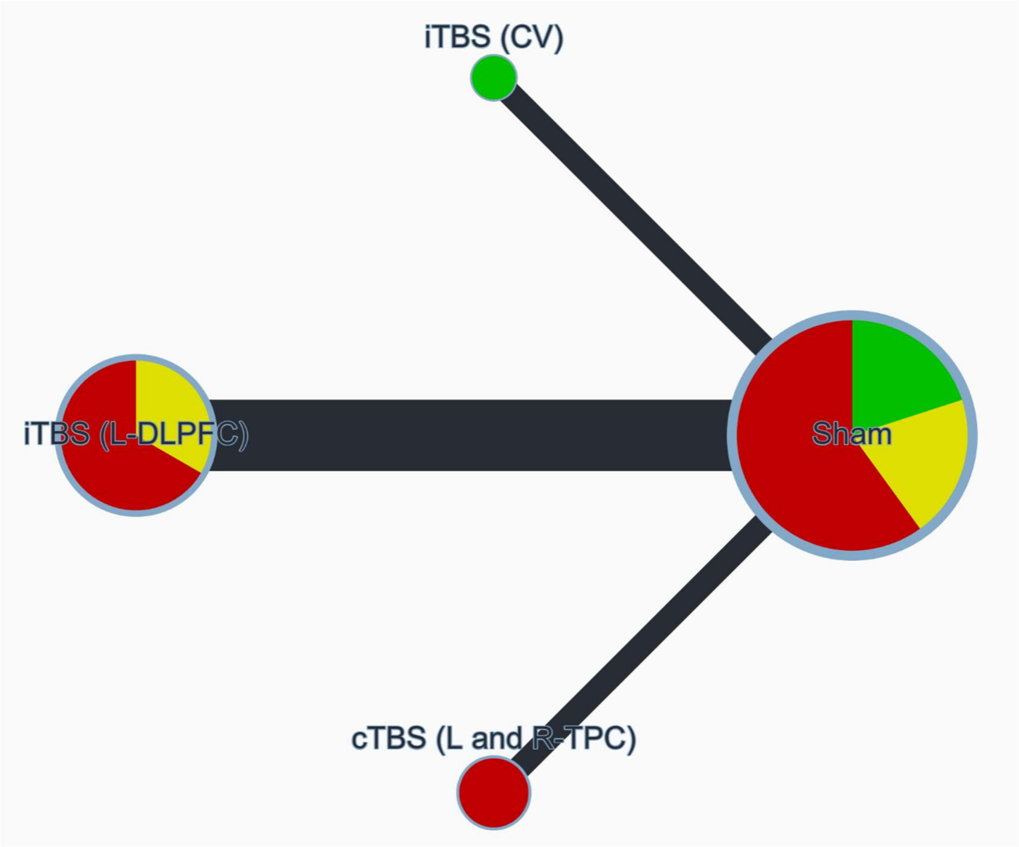

© 2024 Kishi T et al. *JAMA Network Open.*

League table (SMD with 95% confidence interval)

cTBS (L and R-TPC)	0.521 (-0.316, 1.358)	0.602 (-0.029, 1.233)	0.081 (-0.430, 0.591)
	iTBS (CV)	0.081 (-0.678, 0.841)	-0.440 (-1.103, 0.223)
		iTBS (L-DLPFC)	**-0.521 (-0.892, -0.151)**
			Sham

Because lower scores indicate a higher impairment, we reversed the algebraic sign of the numerical scores for these scales.

Comparisons between treatments should be read from left to right, and the estimate is in the cell in common between the column-defining treatment and the row-defining treatment. In the upper right half, SMDs <0 favor the row-defining treatment.

Global heterogeneity

Between study variance (τ^2): 0.000 (heterogeneity assessment: low)

© 2024 Kishi T et al. *JAMA Network Open.*

CINeMA confidence rating

Comparison	Number of studies	Within-study bias	Reporting bias	Indirectness	Imprecision	Heterogeneity	Incoherence	Confidence rating
cTBS (L and R-TPC) vs Sham	1	Major concerns	Some concerns	No concerns	Major concerns	No concerns	Major concerns	Very low
iTBS (CV) vs Sham	1	No concerns	Some concerns	No concerns	Major concerns	No concerns	Major concerns	Very low
iTBS (L-DLPFC) vs Sham	3	Some concerns	Some concerns	No concerns	No concerns	Major concerns	Major concerns	Very low
cTBS (L and R-TPC) vs iTBS (CV)	0	Some concerns	Some concerns	No concerns	Major concerns	No concerns	Major concerns	Very low
cTBS (L and R-TPC) vs iTBS (L-DLPFC)	0	Major concerns	Some concerns	No concerns	Major concerns	No concerns	Major concerns	Very low
iTBS (CV) vs iTBS (L-DLPFC)	0	Some concerns	Some concerns	No concerns	Major concerns	No concerns	Major concerns	Very low

© 2024 Kishi T et al. *JAMA Network Open.*

eAppendix 8. All-Cause Discontinuation

<u>Network meta-analysis</u>

27 studies, 1310 participants

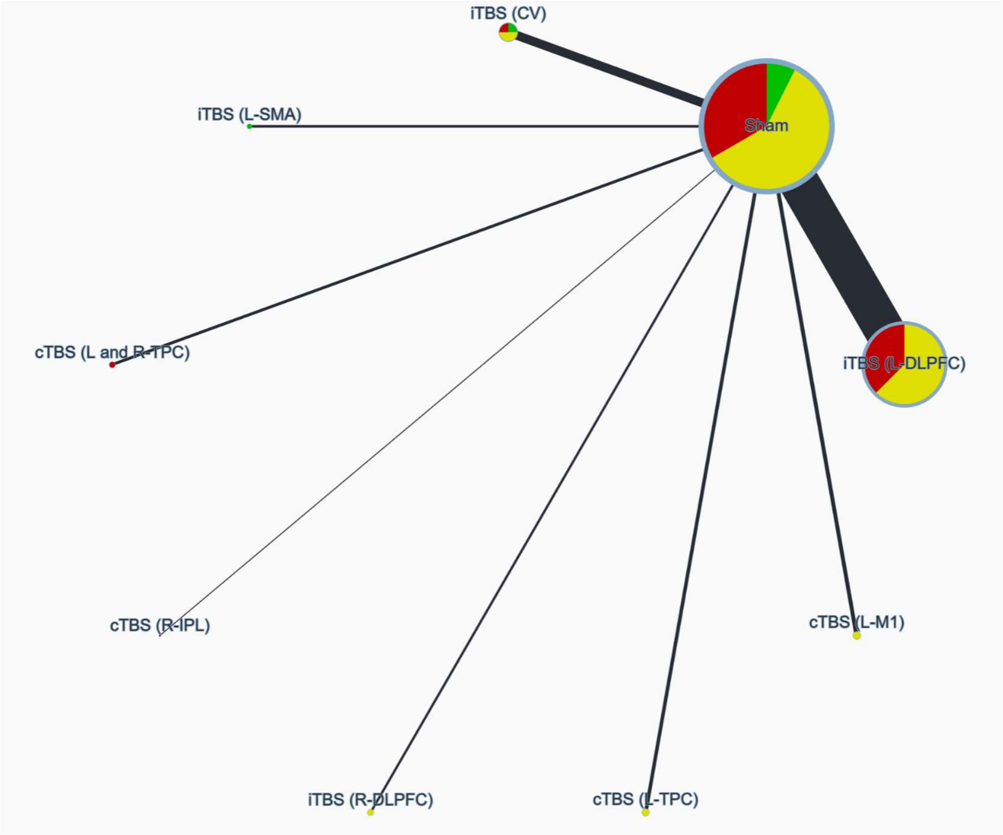

© 2024 Kishi T et al. *JAMA Network Open.*

League table (OR with 95% confidence interval)

cTBS (L and R-TPC)	0.493 (0.043, 5.646)	0.500 (0.054, 4.642)	2.143 (0.175, 26.191)	1.756 (0.245, 12.584)	1.662 (0.351, 7.876)	1.523 (0.239, 9.696)	0.638 (0.069, 5.879)	1.250 (0.300, 5.207)
	cTBS (L-M1)	1.015 (0.074, 13.878)	4.350 (0.251, 75.452)	3.564 (0.324, 39.244)	3.374 (0.425, 26.811)	3.090 (0.309, 30.914)	1.294 (0.095, 17.595)	2.537 (0.351, 18.337)
		cTBS (L-TPC)	4.286 (0.295, 62.240)	3.511 (0.395, 31.206)	3.324 (0.538, 20.522)	3.045 (0.381, 24.339)	1.275 (0.114, 14.256)	2.500 (0.451, 13.843)
			cTBS (R-IPL)	0.819 (0.070, 9.632)	0.776 (0.091, 6.647)	0.711 (0.066, 7.608)	0.298 (0.021, 4.296)	0.583 (0.075, 4.562)
				iTBS (CV)	0.947 (0.213, 4.211)	0.867 (0.144, 5.238)	0.363 (0.041, 3.205)	0.712 (0.183, 2.767)
					iTBS (L-DLPFC)	0.916 (0.242, 3.472)	0.384 (0.063, 2.348)	0.752 (0.405, 1.398)
						iTBS (L-SMA)	0.419 (0.053, 3.322)	0.821 (0.252, 2.670)
							iTBS (R-DLPFC)	1.960 (0.357, 10.753)
								Sham

Comparisons between treatments should be read from left to right and the estimate is in the cell in common between the column-defining treatment and the row-defining treatment. In the upper right half, ORs <1 favour the row-defining treatment.

Global heterogeneity

Between study variance (τ^2): 0.000 (heterogeneity assessment: low)

© 2024 Kishi T et al. *JAMA Network Open.*

CINeMA confidence rating

Comparison	Number of studies	Within-study bias	Reporting bias	Indirectness	Imprecision	Heterogeneity	Incoherence	Confidence rating
cTBS (L and R-TPC) vs Sham	1	Major concerns	Some concerns	No concerns	Major concerns	No concerns	Major concerns	Very low
cTBS (L-M1) vs Sham	2	Some concerns	Some concerns	No concerns	Major concerns	No concerns	Major concerns	Very low
cTBS (L-TPC) vs Sham	1	Some concerns	Some concerns	No concerns	Major concerns	No concerns	Major concerns	Very low
cTBS (R-IPL) vs Sham	1	Major concerns	Some concerns	No concerns	Major concerns	No concerns	Major concerns	Very low
iTBS (CV) vs Sham	4	Some concerns	Some concerns	No concerns	Major concerns	No concerns	Major concerns	Very low
iTBS (L-DLPFC) vs Sham	16	Some concerns	No concerns	No concerns	Major concerns	No concerns	Major concerns	Low
iTBS (L-SMA) vs Sham	1	No concerns	Some concerns	No concerns	Major concerns	No concerns	Major concerns	Very low
iTBS (R-DLPFC) vs Sham	1	Some concerns	Some concerns	No concerns	Major concerns	No concerns	Major concerns	Very low
cTBS (L and R-TPC) vs cTBS (L-M1)	0	Major concerns	Some concerns	No concerns	Major concerns	No concerns	Major concerns	Very low
cTBS (L and R-TPC) vs cTBS (L-TPC)	0	Major concerns	Some concerns	No concerns	Major concerns	No concerns	Major concerns	Very low
cTBS (L and R-TPC) vs cTBS (R-IPL)	0	Major concerns	Some concerns	No concerns	Major concerns	No concerns	Major concerns	Very low
cTBS (L and R-TPC) vs iTBS (CV)	0	Some concerns	Some concerns	No concerns	Major concerns	No concerns	Major concerns	Very low
cTBS (L and R-TPC) vs iTBS (L-DLPFC)	0	Major concerns	Some concerns	No concerns	Major concerns	No concerns	Major concerns	Very low
cTBS (L and R-TPC) vs iTBS (L-SMA)	0	Some concerns	Some concerns	No concerns	Major concerns	No concerns	Major concerns	Very low
cTBS (L and R-TPC) vs iTBS (R-DLPFC)	0	Major concerns	Some concerns	No concerns	Major concerns	No concerns	Major concerns	Very low
cTBS (L-M1) vs cTBS (L-TPC)	0	Some concerns	Some concerns	No concerns	Major concerns	No concerns	Major concerns	Very low
cTBS (L-M1) vs cTBS (R-IPL)	0	Major concerns	Some concerns	No concerns	Major concerns	No concerns	Major concerns	Very low
cTBS (L-M1) vs iTBS (CV)	0	Some concerns	Some concerns	No concerns	Major concerns	No concerns	Major concerns	Very low
cTBS (L-M1) vs iTBS (L-DLPFC)	0	Some concerns	Some concerns	No concerns	Major concerns	No concerns	Major concerns	Very low
cTBS (L-M1) vs iTBS (L-SMA)	0	Some concerns	Some concerns	No concerns	Major concerns	No concerns	Major concerns	Very low
cTBS (L-M1) vs iTBS (R-DLPFC)	0	Some concerns	Some concerns	No concerns	Major concerns	No concerns	Major concerns	Very low
cTBS (L-TPC) vs cTBS (R-IPL)	0	Major concerns	Some concerns	No concerns	Major concerns	No concerns	Major concerns	Very low
cTBS (L-TPC) vs iTBS (CV)	0	Some concerns	Some concerns	No concerns	Major concerns	No concerns	Major concerns	Very low
cTBS (L-TPC) vs iTBS (L-DLPFC)	0	Some concerns	Some concerns	No concerns	Major concerns	No concerns	Major concerns	Very low
cTBS (L-TPC) vs iTBS (L-SMA)	0	Some concerns	Some concerns	No concerns	Major concerns	No concerns	Major concerns	Very low
cTBS (L-TPC) vs iTBS (R-DLPFC)	0	Some concerns	Some concerns	No concerns	Major concerns	No concerns	Major concerns	Very low

© 2024 Kishi T et al. *JAMA Network Open.*

cTBS (R-IPL) vs iTBS (CV)	0	Some concerns	Some concerns	No concerns	Major concerns	No concerns	Major concerns	Very low
cTBS (R-IPL) vs iTBS (L-DLPFC)	0	Major concerns	Some concerns	No concerns	Major concerns	No concerns	Major concerns	Very low
cTBS (R-IPL) vs iTBS (L-SMA)	0	Some concerns	Some concerns	No concerns	Major concerns	No concerns	Major concerns	Very low
cTBS (R-IPL) vs iTBS (R-DLPFC)	0	Major concerns	Some concerns	No concerns	Major concerns	No concerns	Major concerns	Very low
iTBS (CV) vs iTBS (L-DLPFC)	0	Some concerns	Some concerns	No concerns	Major concerns	No concerns	Major concerns	Very low
iTBS (CV) vs iTBS (L-SMA)	0	No concerns	Some concerns	No concerns	Major concerns	No concerns	Major concerns	Very low
iTBS (CV) vs iTBS (R-DLPFC)	0	Some concerns	Some concerns	No concerns	Major concerns	No concerns	Major concerns	Very low
iTBS (L-DLPFC) vs iTBS (L-SMA)	0	Some concerns	Some concerns	No concerns	Major concerns	No concerns	Major concerns	Very low
iTBS (L-DLPFC) vs iTBS (R-DLPFC)	0	Some concerns	Some concerns	No concerns	Major concerns	No concerns	Major concerns	Very low
iTBS (L-SMA) vs iTBS (R-DLPFC)	0	Some concerns	Some concerns	No concerns	Major concerns	No concerns	Major concerns	Very low

© 2024 Kishi T et al. *JAMA Network Open.*

eAppendix 9. Discontinuation Due to Adverse Events

<u>Network meta-analysis</u>

6 studies, 307 participants

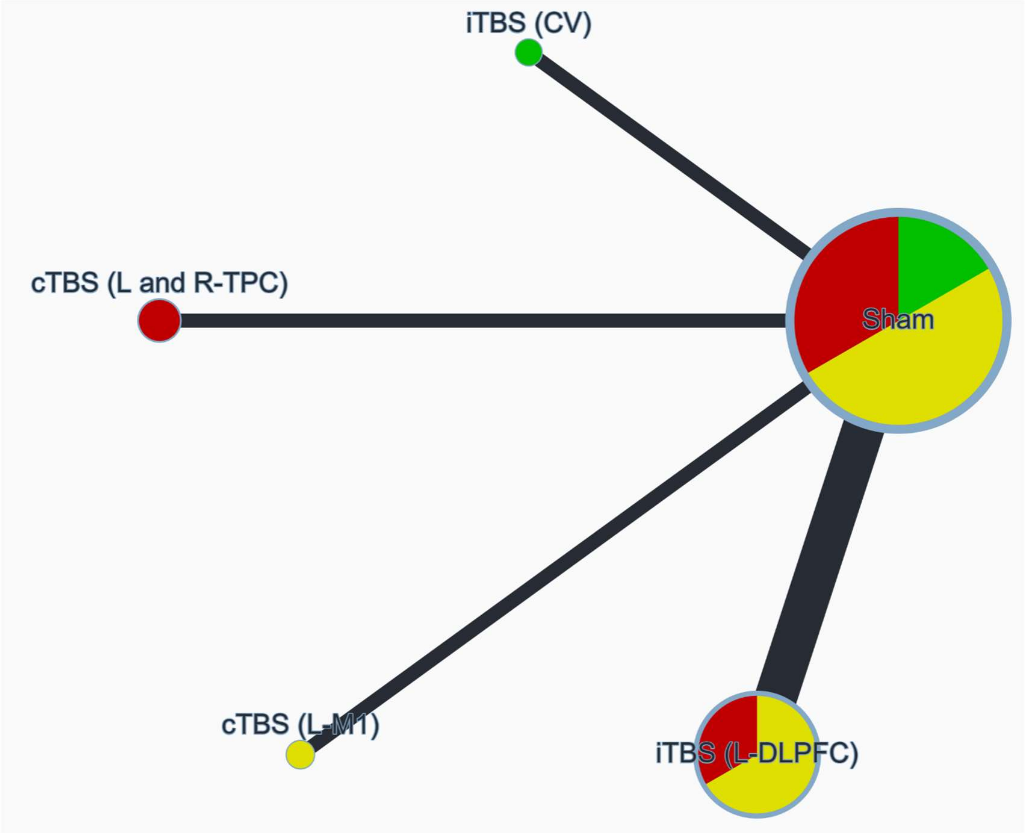

© 2024 Kishi T et al. *JAMA Network Open.*

League table (OR with 95% confidence interval)

cTBS (L and R-TPC)	0.307 (0.002, 51.496)	1.078 (0.004, 292.598)	1.430 (0.018, 113.534)	0.967 (0.019, 50.355)
	cTBS (L-M1)	3.514 (0.021, 599.442)	4.665 (0.109, 200.357)	3.154 (0.121, 82.163)
		iTBS (CV)	1.327 (0.016, 107.286)	0.897 (0.017, 47.679)
			iTBS (L-DLPFC)	0.676 (0.104, 4.403)
				Sham

Comparisons between treatments should be read from left to right and the estimate is in the cell in common between the column-defining treatment and the row-defining treatment. In the upper right half, ORs <1 favour the row-defining treatment.

Global heterogeneity

Between study variance (τ^2): 0.000 (heterogeneity assessment: low)

© 2024 Kishi T et al. *JAMA Network Open.*

CINeMA confidence rating

Comparison	Number of studies	Within-study bias	Reporting bias	Indirectness	Imprecision	Heterogeneity	Incoherence	Confidence rating
cTBS (L and R-TPC) vs Sham	1	Major concerns	Some concerns	No concerns	Major concerns	No concerns	Major concerns	Very low
cTBS (L-M1) vs Sham	1	Some concerns	Some concerns	No concerns	Major concerns	No concerns	Major concerns	Very low
iTBS (CV) vs Sham	1	No concerns	Some concerns	No concerns	Major concerns	No concerns	Major concerns	Very low
iTBS (L-DLPFC) vs Sham	3	Some concerns	Some concerns	No concerns	Major concerns	No concerns	Major concerns	Very low
cTBS (L and R-TPC) vs cTBS (L-M1)	0	Major concerns	Some concerns	No concerns	Major concerns	No concerns	Major concerns	Very low
cTBS (L and R-TPC) vs iTBS (CV)	0	Some concerns	Some concerns	No concerns	Major concerns	No concerns	Major concerns	Very low
cTBS (L and R-TPC) vs iTBS (L-DLPFC)	0	Major concerns	Some concerns	No concerns	Major concerns	No concerns	Major concerns	Very low
cTBS (L-M1) vs iTBS (CV)	0	Some concerns	Some concerns	No concerns	Major concerns	No concerns	Major concerns	Very low
cTBS (L-M1) vs iTBS (L-DLPFC)	0	Some concerns	Some concerns	No concerns	Major concerns	No concerns	Major concerns	Very low
iTBS (CV) vs iTBS (L-DLPFC)	0	Some concerns	Some concerns	No concerns	Major concerns	No concerns	Major concerns	Very low

© 2024 Kishi T et al. *JAMA Network Open.*

eAppendix 10. Headache

Network meta-analysis

15 studies, 795 participants

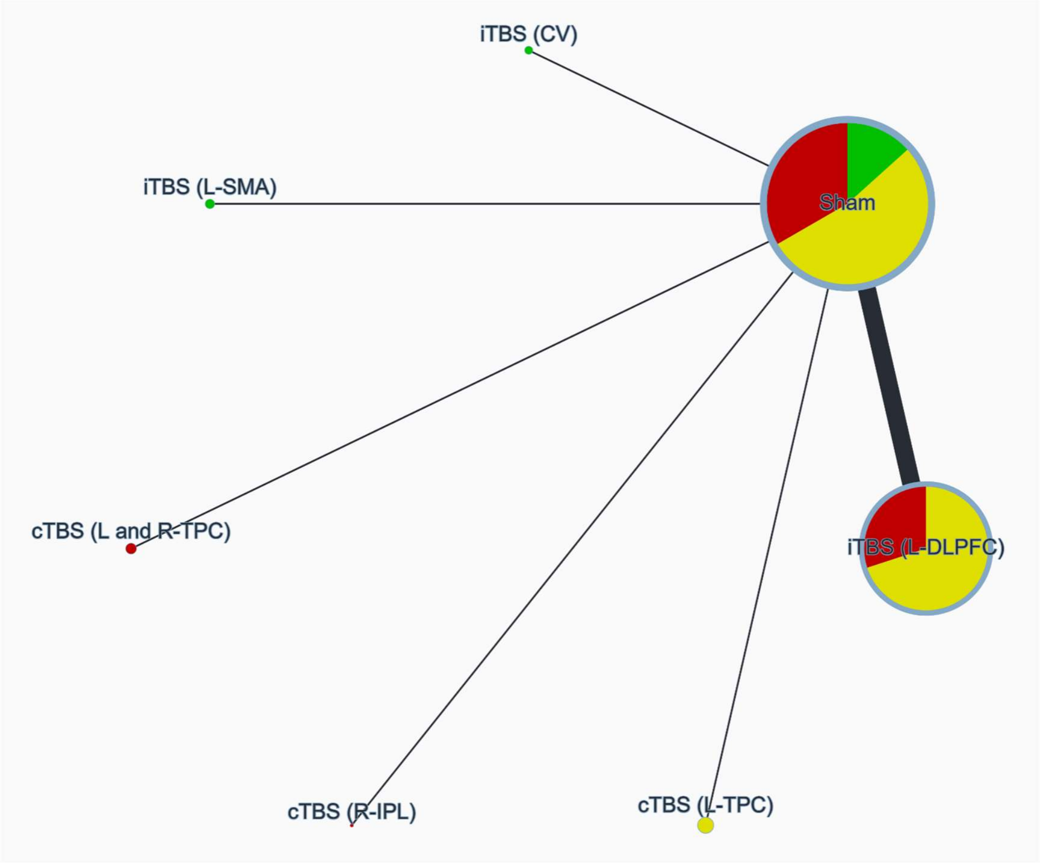

© 2024 Kishi T et al. *JAMA Network Open.*

League table (OR with 95% confidence interval)

cTBS (L and R-TPC)	1.219 (0.217, 6.850)	1.575 (0.105, 23.712)	0.490 (0.049, 4.922)	1.153 (0.235, 5.668)	0.350 (0.048, 2.574)	1.312 (0.308, 5.597)
	cTBS (L-TPC)	1.292 (0.109, 15.353)	0.402 (0.053, 3.042)	0.946 (0.301, 2.969)	0.287 (0.055, 1.509)	1.077 (0.422, 2.746)
		cTBS (R-IPL)	0.311 (0.017, 5.712)	0.732 (0.068, 7.941)	0.222 (0.015, 3.208)	0.833 (0.084, 8.240)
			iTBS (CV)	2.353 (0.348, 15.908)	0.714 (0.075, 6.830)	2.679 (0.445, 16.113)
				iTBS (L-DLPFC)	0.303 (0.066, 1.387)	1.138 (0.590, 2.196)
					iTBS (L-SMA)	3.750 (0.952, 14.764)
						Sham

Comparisons between treatments should be read from left to right and the estimate is in the cell in common between the column-defining treatment and the row-defining treatment. In the upper right half, ORs <1 favour the row-defining treatment.

Global heterogeneity

Between study variance (τ^2): 0.000 (heterogeneity assessment: low)

© 2024 Kishi T et al. *JAMA Network Open.*

CINeMA confidence rating

Comparison	Number of studies	Within-study bias	Reporting bias	Indirectness	Imprecision	Heterogeneity	Incoherence	Confidence rating
cTBS (L and R-TPC) vs Sham	1	Major concerns	Some concerns	No concerns	Major concerns	No concerns	Major concerns	Very low
cTBS (L-TPC) vs Sham	1	Some concerns	Some concerns	No concerns	Major concerns	No concerns	Major concerns	Very low
cTBS (R-IPL) vs Sham	1	Major concerns	Some concerns	No concerns	Major concerns	No concerns	Major concerns	Very low
iTBS (CV) vs Sham	1	No concerns	Some concerns	No concerns	Major concerns	No concerns	Major concerns	Very low
iTBS (L-DLPFC) vs Sham	10	Some concerns	No concerns	No concerns	Major concerns	No concerns	Major concerns	Low
iTBS (L-SMA) vs Sham	1	No concerns	Some concerns	No concerns	Major concerns	No concerns	Major concerns	Very low
cTBS (L and R-TPC) vs cTBS (L-TPC)	0	Major concerns	Some concerns	No concerns	Major concerns	No concerns	Major concerns	Very low
cTBS (L and R-TPC) vs cTBS (R-IPL)	0	Major concerns	Some concerns	No concerns	Major concerns	No concerns	Major concerns	Very low
cTBS (L and R-TPC) vs iTBS (CV)	0	Some concerns	Some concerns	No concerns	Major concerns	No concerns	Major concerns	Very low
cTBS (L and R-TPC) vs iTBS (L-DLPFC)	0	Major concerns	Some concerns	No concerns	Major concerns	No concerns	Major concerns	Very low
cTBS (L and R-TPC) vs iTBS (L-SMA)	0	Some concerns	Some concerns	No concerns	Major concerns	No concerns	Major concerns	Very low
cTBS (L-TPC) vs cTBS (R-IPL)	0	Major concerns	Some concerns	No concerns	Major concerns	No concerns	Major concerns	Very low
cTBS (L-TPC) vs iTBS (CV)	0	Some concerns	Some concerns	No concerns	Major concerns	No concerns	Major concerns	Very low
cTBS (L-TPC) vs iTBS (L-DLPFC)	0	Some concerns	Some concerns	No concerns	Major concerns	No concerns	Major concerns	Very low
cTBS (L-TPC) vs iTBS (L-SMA)	0	Some concerns	Some concerns	No concerns	Major concerns	No concerns	Major concerns	Very low
cTBS (R-IPL) vs iTBS (CV)	0	Some concerns	Some concerns	No concerns	Major concerns	No concerns	Major concerns	Very low
cTBS (R-IPL) vs iTBS (L-DLPFC)	0	Major concerns	Some concerns	No concerns	Major concerns	No concerns	Major concerns	Very low
cTBS (R-IPL) vs iTBS (L-SMA)	0	Some concerns	Some concerns	No concerns	Major concerns	No concerns	Major concerns	Very low
iTBS (CV) vs iTBS (L-DLPFC)	0	Some concerns	Some concerns	No concerns	Major concerns	No concerns	Major concerns	Very low
iTBS (CV) vs iTBS (L-SMA)	0	No concerns	Some concerns	No concerns	Major concerns	No concerns	Major concerns	Very low
iTBS (L-DLPFC) vs iTBS (L-SMA)	0	Some concerns	Some concerns	No concerns	Major concerns	No concerns	Major concerns	Very low

© 2024 Kishi T et al. *JAMA Network Open.*

eAppendix 11. Dizziness

<u>Network meta-analysis</u>

7 studies, 407 participants

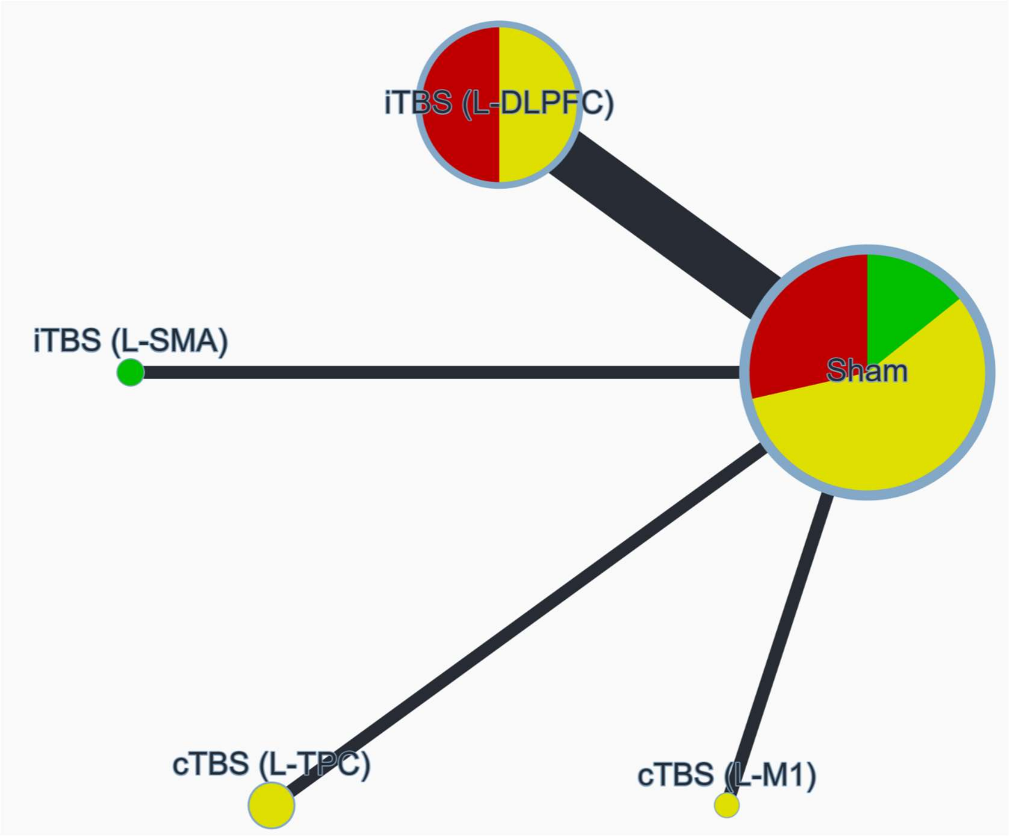

© 2024 Kishi T et al. *JAMA Network Open.*

League table (OR with 95% confidence interval)

cTBS (L-M1)	1.865 (0.061, 56.633)	2.776 (0.087, 88.173)	2.241 (0.059, 85.773)	3.154 (0.121, 82.163)
	cTBS (L-TPC)	1.488 (0.321, 6.900)	1.201 (0.177, 8.178)	1.691 (0.615, 4.646)
		iTBS (L-DLPFC)	0.807 (0.110, 5.945)	1.136 (0.358, 3.601)
			iTBS (L-SMA)	1.407 (0.276, 7.182)
				Sham

Comparisons between treatments should be read from left to right and the estimate is in the cell in common between the column-defining treatment and the row-defining treatment. In the upper right half, ORs <1 favour the row-defining treatment.

Global heterogeneity

Between study variance (τ^2): 0.000 (heterogeneity assessment: low)

© 2024 Kishi T et al. *JAMA Network Open.*

CINeMA confidence rating

Comparison	Number of studies	Within-study bias	Reporting bias	Indirectness	Imprecision	Heterogeneity	Incoherence	Confidence rating
cTBS (L-M1) vs Sham	1	Some concerns	Some concerns	No concerns	Major concerns	No concerns	Major concerns	Very low
cTBS (L-TPC) vs Sham	1	Some concerns	Some concerns	No concerns	Major concerns	No concerns	Major concerns	Very low
iTBS (L-DLPFC) vs Sham	4	Some concerns	Some concerns	No concerns	Major concerns	No concerns	Major concerns	Very low
iTBS (L-SMA) vs Sham	1	No concerns	Some concerns	No concerns	Major concerns	No concerns	Major concerns	Very low
cTBS (L-M1) vs cTBS (L-TPC)	0	Some concerns	Some concerns	No concerns	Major concerns	No concerns	Major concerns	Very low
cTBS (L-M1) vs iTBS (L-DLPFC)	0	Some concerns	Some concerns	No concerns	Major concerns	No concerns	Major concerns	Very low
cTBS (L-M1) vs iTBS (L-SMA)	0	Some concerns	Some concerns	No concerns	Major concerns	No concerns	Major concerns	Very low
cTBS (L-TPC) vs iTBS (L-DLPFC)	0	Some concerns	Some concerns	No concerns	Major concerns	No concerns	Major concerns	Very low
cTBS (L-TPC) vs iTBS (L-SMA)	0	Some concerns	Some concerns	No concerns	Major concerns	No concerns	Major concerns	Very low
iTBS (L-DLPFC) vs iTBS (L-SMA)	0	Some concerns	Some concerns	No concerns	Major concerns	No concerns	Major concerns	Very low

© 2024 Kishi T et al. *JAMA Network Open.*
