# Supplementary material for: Theta Burst Stimulation Protocols for Schizophrenia: A Systematic Review and Network Meta-Analysis
Source: JAMA Netw Open. 2024 Oct 24;7(10):e2441159. doi: 10.1001/jamanetworkopen.2024.41159 (PMC11581676; doi:10.1001/jamanetworkopen.2024.41159)
Supplement: Supplement 2. — Data Sharing Statement [file jamanetwopen-e2441159-s002.pdf]

Data Sharing Statement

Kishi. Comparative Efficacy and Acceptability of Theta Burst Stimulation Protocols for Schizophrenia. *JAMA Netw Open*. Published October 24, 2024.
doi:10.1001/jamanetworkopen.2024.41159

Data
Data available: Yes
Data types: Other (please specify)
Additional Information: Data extracted from RCTs will be made available upon reasonable request to the corresponding author.
How to access data: Data extracted from RCTs will be made available upon reasonable request to the corresponding author.
When available: With publication

Supporting Documents
Document types: None

Additional Information
Who can access the data: Data extracted from RCTs will be made available upon reasonable request to the corresponding author.
Types of analyses: Data extracted from RCTs will be made available upon reasonable request to the corresponding author.
Mechanisms of data availability: Data extracted from RCTs will be made available upon reasonable request to the corresponding author.
